# Substrate specificity and protein stability drive the divergence of plant-specific DNA methyltransferases

**DOI:** 10.1126/sciadv.adr2222

**Published:** 2024-11-06

**Authors:** Jianjun Jiang, Jia Gwee, Jian Fang, Sarah M. Leichter, Dean Sanders, Xinrui Ji, Jikui Song, Xuehua Zhong

**Affiliations:** ^1^ Wisconsin Institute for Discovery and Laboratory of Genetics, University of Wisconsin-Madison, Madison, WI 53715, USA.; ^2^ State Key Laboratory of Crop Stress Adaptation and Improvement, Academy for Advanced Interdisplinary Studies and The Zhongzhou Laboratory for Integrative Biology, Henan University, Zhengzhou, Henan 450000, China.; ^3^ Department of Biology, Washington University in St. Louis, St. Louis, MO 63130, USA.; ^4^ Department of Biochemistry, University of California, Riverside, CA 92521, USA.

## Abstract

DNA methylation is an important epigenetic mechanism essential for transposon silencing and genome integrity. Across evolution, the substrates of DNA methylation have diversified between kingdoms. In plants, chromomethylase3 (CMT3) and CMT2 mediate CHG and CHH methylation, respectively. However, how these two methyltransferases diverge on substrate specificities during evolution remains unknown. Here, we reveal that CMT2 originates from a duplication of an evolutionarily ancient CMT3 in flowering plants. Lacking a key arginine residue recognizing CHG in CMT2 impairs its CHG methylation activity in most flowering plants. An engineered V1200R mutation empowers CMT2 to restore CHG and CHH methylations in *Arabidopsis cmt2cmt3* mutant, testifying a loss-of-function effect for CMT2 during evolution. CMT2 has evolved a long and unstructured amino terminus critical for protein stability, especially under heat stress, and is plastic to tolerate various natural mutations. Together, this study reveals the mechanism of chromomethylase divergence for context-specific DNA methylation in plants and sheds important lights on DNA methylation evolution and function.

## Teaser

Functional diversification of substrate-specific DNA methyltransferases accounts for plant genome complexity during evolution.

## INTRODUCTION

DNA methylation is an important gene regulatory mechanism and plays critical roles in many biological processes such as development, transposon silencing, and genome integrity (*1*, *2*). Dysregulation of DNA methylation can lead to pleiotropic developmental defects in plants and the development of diseases such as cancer in mammals (*3*, *4*). While most methylated DNA in mammals is found in the CG context, DNA methylation in plants occurs in CG, CHG, and CHH (H = A, T, C). In *Arabidopsis thaliana*, this complex nature of substrate preference is facilitated by multiple DNA methyltransferases including DOMAINS REARRANGED METHYLTRANSFERASE 2 (DRM2), METHYLTRANSFERASE 1 (MET1), CHROMOMETHYLASE 3 (CMT3), and CMT2 (*5*, *6*).

Chromomethylases are plant-specific DNA methyltransferases containing a chromo domain, a bromo-adjacent homology (BAH) domain, and a catalytic methyltransferase domain (*7*, *8*). In *A. thaliana*, there are three CMT genes, *CMT1*, *CMT2*, and *CMT3*, that emerged from duplication events through the evolution of green plants (*9*). Genome duplication events such as whole genome duplication and small-scale duplication are abundant in plants and are thought to be a driving force for diversity and speciation (*10*). While most duplicated genes become silenced or pseudogenes, functional diversifications such as subfunctionalization and neofunctionalization serve as potential mechanism behind duplicate retention (*11*). In the case of *A. thaliana*, the *CMT1* gene is dispensable for DNA methylation, while CMT2 and CMT3 appear to have diversified for the labor division for non-CG methylation (*12*–*14*). However, how the two CMTs have diversified to confer the increasing complexity of non-CG methylation during plant evolution remains unknown.

Functionally, CMT3 maintains symmetric CHG methylation on transposable elements (TEs) and repetitive sequences in the heterochromatin (*13*, *14*). Recent studies have also shown that CMT3 is involved in the de novo establishment of gene-body methylation (*9*, *15*, *16*). On the other hand, CMT2 maintains the asymmetric CHH methylation alongside DRM2, with CMT2 methylating DNA within long TEs in heterochromatic regions whereas DRM2 mediates methylation within short TEs and at the edges of long TEs (*13*). CHH methylation plays important roles in TE silencing and environmental adaptation, and both CMT2 and DRM2 pathways enable a “double-lock mechanism,” indicated by the conversion of CMT2 targets to DRM2 targets during the loss of remodeler DDM1, for ensuring maintenance of CHH methylation and genome integrity (*17*–*19*). CMT2 is also capable of maintaining CHG methylation alongside CMT3, suggesting a partial redundancy between the two chromomethylases to ensure maintenance of methylation in the heterochromatin for genome stability (*14*).

Although CMT2 and CMT3 have distinct DNA substrate preferences, both proteins recognize and bind to methylated histone 3 lysine 9 (H3K9me) through both chromo and BAH domains to methylate DNA substrate (*12*, *14*, *20*). The substrate specificity of CMT3 has recently been illustrated in its functional homolog in maize, ZMET2, where the enzyme is activated by allosteric recognition of H3K9me2 and histone 3 lysine 18, and the base-specific interactions of the methyltransferase domain with the hemimethylated CHG site and deformation of the DNA around the target cytosine for methylation (*21*).

CMTs are present in major green plant lineages ranging from green algae to angiosperms. *Physcomitrella patens* CMT (PpCMT) from the homologous β (hCMTβ) clade can methylate DNA in the CHG context, suggesting that CHG methylation is a conserved function of CMTs predating angiosperm CMTs (*22*, *23*). In angiosperms, CMTs are further evolved via whole genome duplication event resulting in the CMT2 subclade and the CMT1/3 subclade in eudicots, ZMET subclade in monocots and magnoliids, and CMT subclade in *Amborella trichopoda* (*9*). However, not all angiosperm species have both CMT2 and CMT3; *Zea mays* lost CMT2 although its close relative *Sorghum bicolor* retains it, while two close Brassicaceae relatives of *A. thaliana*, *Eutrema salsugineum* and *Conringia planisiliqua*, lost their CMT3 (*9*, *13*, *16*). Furthermore, some angiosperm species such as *Oryza sativa* contain multiple copies of CMT3 (*24*), yet the basis and consequence behind the variation and retention of CMTs in various angiosperm species remain unknown.

Here, we investigated the molecular mechanism underlying the divergence of CMT2 and CMT3 by carrying out a comprehensive structural, functional, and evolutionary study. We noted that an arginine residue crucial for the recognition of CHG by CMT3 (R745) showed great variations in CMT2, explaining its loss of CHG specificity. Mutation of the corresponding residue in CMT2 to arginine in *Arabidopsis* (V1200R) gained CHG methylation activity and resilenced a subset of TEs in *cmt2 cmt3* mutant with CMT3-like function. While CMT3 has a short N terminus, CMT2 contains a long and disordered N terminus, which is a common characteristic among many plant species. This long N terminus regulated CMT2 stability and mediated heat-induced CMT2 degradation. Furthermore, CMT2 N terminus is more plastic and tolerant to mutations as various CMT2 variations at the N terminus are observed in nature. Together, this study reveals the mechanism of chromomethylase divergence and provides important insights into DNA methylation function and evolution in plants.

## RESULTS

### CMT2 is duplicated from CMT3

**Fig. 1.**
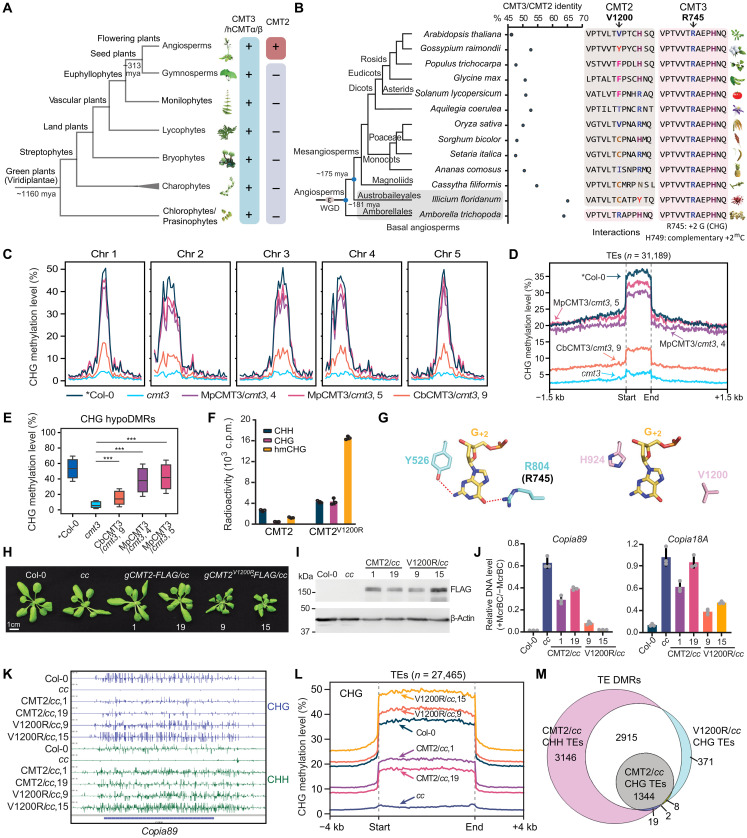
Loss of an arginine residue confers CMT2 a CMT3-distinct substrate preference. (**A**) A phylogenetic tree depicting the presence of CMT3 (or its close homologs hCMTα/β) and CMT2 in green plants (*Viridiplantae*). (**B**) The protein sequence identity between CMT2 and CMT3 (left) and the residue contacting +2 guanine (G_+2_) and corresponding residue in CMT2 (right) in flowering plants. (**C**) Metaplots showing CHG methylation levels by *M. polymorpha* CMT3 (MpCMT3) and *C. braunii* CMT3 (CbCMT3) in *cmt3* mutant background. *Col-0 from GSE39901. (**D**) Metaplots showing the average CHG methylation over all TEs in *Arabidopsis.* (**E**) Boxplots showing CHG methylation levels over CHG hypoDMRs. ***P < 0.001. (**F**) In vitro DNA methyltransferase assay of CMT2 and CMT2^V1200R^ on CHH, CHG, or hemimethylated CHG (hmCHG) DNA substrates. (**G**) Structure of Maize ZMET2-DNA complex (Protein Data Bank 7UBU) showing the interaction between ZMET2 Y526 and R804 and the guanine at the +2 position (G_+2_) from the target cytosine in a CHG context. The ZMET2 R804-corresponding residue in CMT3, R745, is shown in parentheses (left). Structural model of *Arabidopsis* CMT2 showing the position of G_+2_ and its surrounding residues, V1200 and H924 (right). (**H**) Phenotype images of two independent transgenic CMT2 and CMT2^V1200R^ plants in *cmt2cmt3* (*cc*) mutant background. (**I**) Immunoblots showing CMT2 and CMT2^V1200R^ protein levels from the plants in (H). Actin serves as a loading control. (**J**) McrBC–quantitative PCR (qPCR) assay determining DNA methylation levels of two CMT2-targeted TEs, *Copia89* and *Copia18A*. (**K**) Genome browser view of CHG and CHH methylation levels of *Copia89* from BS-seq data. (**L**) Metaplots showing the average CHG methylation over all TEs in *Arabidopsis*. (**M**) Venn diagram showing the overlapping of DMRs identified by comparing indicated genotypes with Col-0. mya, million years ago; c.p.m., counts per minute.

Our recent structural and functional study of maize CMT3 (ZMET2) homolog has revealed critical residues responsible for its enzymatic preference for CHG substrates (*21*). Using these key residues as a signature, we performed a phylogenetic analysis of CMT3 and CMT2 from a range of species representing major plant lineages across the evolution (data S1). We found that CMT3 or its close homologs hCMTα/β is present in all green plants (Viridiplantae), including streptophytes, chlorophytes, and prasinophytes, whereas CMT2 only appears in flowering plants (Fig. 1A and data S1), consistent with previous reports (*9*, *23*). Key residues of Y776, R804, and H808 for CHG substrate recognition and W224, Y302, and M641 for H3 binding in ZMET2 are highly conserved across streptophytes, including charophytes and land plants, but undergo notable variations in chlorophyte green algae (Fig. 1B and fig. S1A), suggesting an early emergence of CMT3 as a conserved DNA methyltransferase in green plants. All but one of these ZMET2 residues are also conserved in angiosperm CMT2 (fig. S1B). These data suggest that the ancestor of CMT2/3 and hCMTα/β resembles CMT3, thus we designated the ancient CMT as CMT3 for simplicity in subsequent discussions.

To examine whether CMT3 homologs from the early diverged plant species remain functional in flowering plants, we ectopically expressed *UBQ10* promoter–driven coding sequences of CMT3 from a charophyte species (*Chara braunii*, CbCMT3) and a bryophyte species (*Marchantia polymorpha*, MpCMT3) and transformed them into *Arabidopsis cmt3* mutant (fig. S2, A and B). We performed a McrBC (an enzyme that specifically cleaves methylated DNA) digestion assay and found that MpCMT3 was able to partially methylate DNA on *Cluster4* (a well-known CMT3 target site), while CbCMT3 showed little methylation activity (fig. S2C). Next, we performed genome-wide bisulfite sequencing (BS-seq) and found that MpCMT3 nearly complemented *cmt3* showing high levels of CHG methylation comparable to wild-type Columbia-0 (Col-0), while CbCMT3 showed low activity in CHG methylation (Fig. 1, C to E, and fig. S2D). We then compared the differentially methylated regions (DMRs) and found that the majority of CHG methylation recovered regions by these CMT3 homologs were original CMT3 targets (fig. S2E), suggesting that ancient CMTs functionally resemble CMT3.

CMT2 is paralogous to CMT3 and shares high protein sequence similarity, especially in basal angiosperms (Fig. 1B). They demonstrate distinct substrate preference for DNA methylation. While CMT3 preferentially methylates hemimethylated CHG (*20*, *21*), CMT2 exhibited a considerably higher methylation activity on CHH over CHG DNA substrate (Fig. 1F and fig. S3A). In addition, CMT2 showed a modest (~2-fold) substrate preference for hemimethylated over unmethylated CHG DNA (Fig. 1F and fig. S3A), suggesting a residual maintenance methyltransferase activity of CMT2 for CHG sites, in line with previous reports of redundancy (*14*, *17*). To further understand the CMT2 evolution, we performed a similar phylogenetic analysis and found that CMT2 from basal angiosperms *A. trichopoda* contains the same arginine in the equivalent to CMT3 R745, a key residue for CHG substrate recognition (Fig. 1B). However, this arginine has diversified into different nonpositively charged residues in higher angiosperm species such as cysteine in *Setaria italica*, phenylalanine in *Glycine max*, and valine in *A. thaliana* (Fig. 1B and fig. S1B). The conservation of this CHG-recognizing arginine residue in basal angiosperms further supports the notion that CMT2 originates from CMT3 duplication, likely along with the whole genome duplication event epsilon (ε) during angiosperms evolution (Fig. 1B) (*25*).

### CMT2 V1200R induces a gain in CHG methylation in vitro and in vivo

To investigate whether this change of CHG-recognizing arginine causes the loss of CHG specificity in CMT2, we first generated a structural model of the CMT2 in complex with hmCHG (fig. S3, B to F) based on the ZMET2 crystal structure. We found that the hydrogen bonding interaction for base-specific recognition of the +2 guanine (G_+2_) in the ZMET2-hmCHG complex was no longer preserved in CMT2, due to the replacement of ZMET2 Y526 and R804 by the corresponding H924 and V1200 of CMT2 (Fig. 1G), explaining the loss of the CHG specificity of CMT2. We then substituted the corresponding V1200 in *A. thaliana* CMT2 with arginine (CMT2^V1200R^). Our in vitro methyltransferase assay showed that the CMT2^V1200R^ mutant has activities about 16-fold higher on hmCHG and 8-fold higher on CHG substrates compared to the wild-type CMT2 (Fig. 1F). In addition, the CMT2^V1200R^ mutation gives rise to a higher activity on the CHH substrate, albeit to a lesser extent (Fig. 1F). To understand the effect of this mutation in vivo, we expressed the genomic fragments of CMT2 and CMT2^V1200R^ fused with a FLAG tag in *cmt2 cmt3* (*cc*) and *drm1 drm2 cmt2 cmt3* (*ddcc*) mutants (Fig. 1, H and I, and fig. S4, A and B). We first investigated the DNA methylation levels of two TEs (*Copia89* and *Copia18A*) cotargeted by CMT2 and CMT3 and found that the introduction of V1200R led to higher methylation than wild-type CMT2 (Fig. 1J and fig. S4C). We next performed BS-seq to investigate the effect of the V1200R mutation on genome-wide methylation levels (table S1). While complementation by CMT2 led to a partial rescue of CHG methylation, introduction of V1200R led to a gain of global CHG methylation with or without the de novo methyltransferase DRM2 (Fig. 1, K and L, and fig. S4, D to G).

To test whether the V1200R mutation could alter CMT2 targeting, we compared the DMRs and examined their genomic locations. We found that most TEs targeted by CMT2^V1200R^ for CHG methylation were original CMT2 targets for CHH methylation (Fig. 1M). To further investigate the chromatin features of these regions that gained CHG methylation by CMT2^V1200R^, we explored their local chromatin environments and locations in the genome. Like CMT2, CMT2^V1200R^ methylated regions contain lower chromatin accessibility (fig. S5, A and B). We also found that the increased CHG methylation regions by CMT2^V1200R^ corresponded to chromatin states annotated for intergenic and centromeric regions (fig. S5C). Together, these data suggested that the CMT2^V1200R^ mutation renders gain of CHG methylation function with minimal effect on chromatin targeting.

### CMT2^V1200R^-mediated CHG methylation represses TEs

**Fig. 2.**
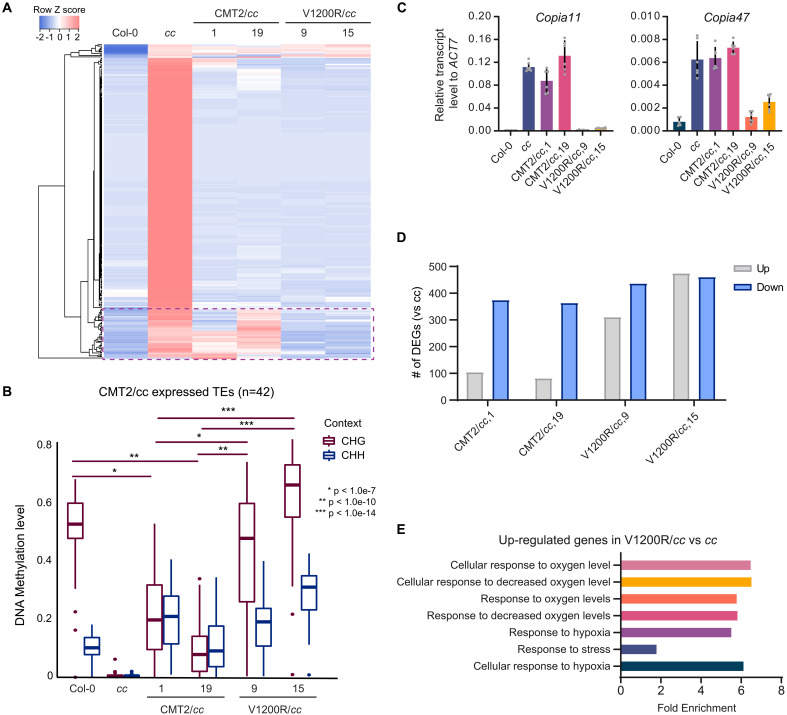
CMT2^V1200R^ represses both CMT2- and CMT3-targeted TEs. (**A**) Heatmaps showing expression levels of up-regulated TEs in two independent CMT2/*cc* and V1200R/*cc* transgenic lines. *cc*, *cmt2cmt3*. The dashed box indicates TEs resilenced by CMT2^V1200R^. (**B**) CHG and CHH DNA methylation levels of TEs remaining expressed in CMT2/*cc*. (**C**) Relative transcript levels of *Copia11* and *Copia47* measured by quantitative reverse transcription PCR (RT-qPCR) in CMT2/*cc* and V1200R/*cc* transgenic plants. The transcript level was normalized to *ACT7*. (**D**) Number of differentially expressed genes (DEGs) in CMT2/*cc* and V1200R/*cc*. (**E**) Gene Ontology (GO) terms of up-regulated genes in V1200R/*cc*.

To understand the transcriptional consequences from the gain of CHG methylation by CMT2^V1200R^, we performed RNA sequencing in CMT2/*cc* and V1200R/*cc* with Col-0 and *cc* as controls (table S2). We noted that a subset of TEs (*n* = 42) up-regulated in *cc* due to hypomethylation were resilenced by the introduction of CMT2^V1200R^ but not CMT2 (Fig. 2A). These TEs were enriched for *LINE*/*L1* and *LTR*/*Copia* type retrotransposons (fig. S6A). We further determined the DNA methylation level in TEs that were specifically resilenced by V1200R/*cc* and found that the CHG methylation levels were comparable to Col-0 in V1200R/*cc*, while CMT2 only partially remethylated these TEs in *cc* (Fig. 2B), suggesting that the low expression in V1200R/*cc* was due to gain of CHG methylation. We next examined the expression of two TEs, *Copia11* and *Copia47*, and found that both TEs were resilenced by CMT2^V1200R^, but not CMT2 (Fig. 2C).

Similarly, we found more differentially expressed genes (DEGs) in the two V1200R/*cc* lines compared with CMT2/*cc* (Fig. 2D). Among the common DEGs of two V1200R/*cc* lines, many of the up-regulated genes (*n* = 269) have low expression levels in Col-0, *cc*, and CMT2/*cc* (fig. S6B). Conversely, lowly expressed genes (*n* = 85) in V1200R/*cc* have higher expression in Col-0, *cc*, and CMT2/*cc* (fig. S6B). Genes up-regulated in only V1200R/*cc* are enriched for Gene Ontology (GO) terms involved in response to stress and decreased oxygen levels (Fig. 2E). Elevated expression of stress-related genes in V1200R lines may help explain the developmentally stunted, smaller plant phenotype (Fig. 1H).

### CMT2 contains a long N terminus important for nuclear localization

**Fig. 3.**
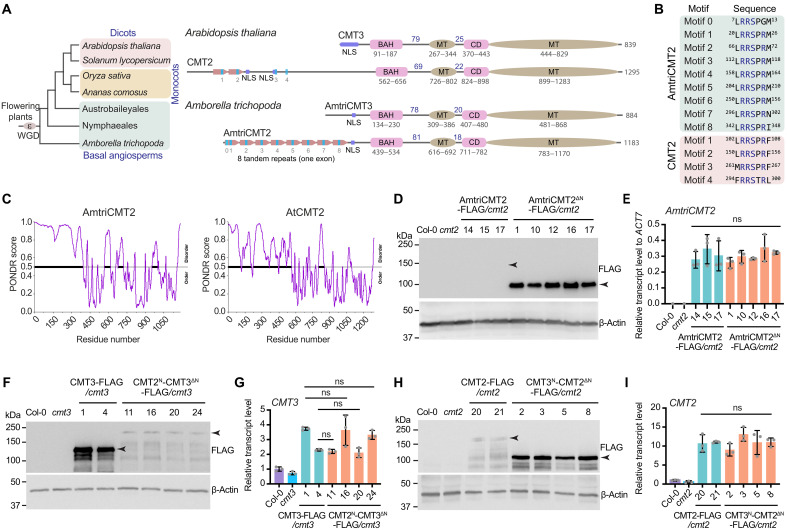
The long CMT2 N terminus is disordered and controls protein level. (**A**) Diagrams showing the domain structure of CMT2 and CMT3 from *A. thaliana* (dicots) and *A. trichopoda* (basal angiosperms). The blue bars indicate tandem repeats. MT, methyltransferase domain; CD, chromo domain. Numbers above lines depict the number of amino acids between domains. WGD, whole genome duplication. (**B**) Alignment of the conserved RRS motifs in CMT2 N terminus of *Amborella* and *Arabidopsis*. (**C**) Graphs of intrinsic disorder scored by PONDR for CMT2 of *Amborella* and *Arabidopsis*. *y* axis indicates PONDR VSL2 scores, and *x* axis indicates the residue positions. (**D**) Immunoblots for protein levels of full-length *Amborella* CMT2 (AmtriCMT2) and N terminus–truncated *Amborella* CMT2 (AmtriCMT2^ΔN^) expressing in *Arabidopsis cmt2* mutant background. Actin serves as a loading control. Numbers represent the independent transgenic lines. (**E**) RT-qPCR showing relativ*e AmtriCMT2* transcript levels in AmtriCMT2 and AmtriCMT2^ΔN^ transgenic lines normalized to *ACT7*. (**F**) Immunoblots for protein levels of wild-type (CMT3) and N-terminal swapped CMT3 (CMT2^N^-CMT3^ΔN^) transgenic lines in *cmt3* mutant. (**G**) RT-qPCR showing relativ*e CMT3* transcript levels in CMT3 and CMT2^N^-CMT3^ΔN^ transgenic plants. Transcript levels were first normalized to *CMT3* level in Col-0 and then to *ACT7* level in Col-0. (**H**) Immunoblots for protein levels of wild-type (CMT2) and N-terminal swapped CMT2 (CMT3^N^-CMT2^ΔN^) transgenic lines in *Arabidopsis cmt2* mutant. (**I**) RT-qPCR showing relativ*e CMT2* transcript levels of CMT2 and CMT3^N^-CMT2^ΔN^ transgenic lines. Transcript levels were first normalized to *CMT2* level in Col-0 and then to *ACT7* level in Col-0. ns, not significant.

Despite sharing similar protein domains, CMT2 and CMT3 differ in their protein lengths with CMT2 having a longer N terminus compared to CMT3 in *Arabidopsis* (i.e., ~550 amino acids in CMT2 and ~90 amino acids in CMT3) (Fig. 3A). Similarly, *Amborella* CMT2 (AmtriCMT2) contains a long N terminus despite sharing similar domain structures with AmtriCMT3 (Fig. 3A). Further analysis revealed a longer N terminus of CMT2 compared to CMT3 in representative angiosperm species that contain both proteins, suggesting a conserved function of this long N terminus (fig. S7A). We found that AmtriCMT2 N terminus contains eight tandem repeats, each encoding conserved “RRSPR” sequences (RRS motif) (Fig. 3B and fig. S7B). This RRS motif is conserved in CMT2 of other angiosperm species, varying from one to nine copies containing RRSxR (x represents any amino acid) (fig. S7C). To investigate how the long N terminus have emerged, we compared the DNA sequences of AmtriCMT2 and AmtriCMT3 and found that the AmtriCMT2 N-terminal sequence bared similarity to AmtriCMT3 promoter sequences (fig. S8A), suggesting that the long N terminus of AmtriCMT2 may arise from exonization of AmtriCMT3 promoter during a duplication event (fig. S8B).

Next, we predicted AmtriCMT2 N-terminal structure and found it to be disordered using Prediction of Natural Disordered Regions (PONDR) (Fig. 3C). The N terminus of both CMT3 and CMT2 is also predicted to contain nuclear localization sequences (NLS) (Fig. 3A and fig. S9A). Upon deletion of the N terminus, we observed a cytosolic localization of CMT3 in both *Nicotiana benthamiana* and *Arabidopsis* (fig. S9, B and C). Similarly, CMT2 N-terminal deletion [CMT2^ΔN^–green fluorescent protein (GFP)] resulted in cytoplasmic localization in both *N. benthamiana* and *Arabidopsis*, whereas the N terminus only (CMT2^N^-GFP) showed nuclear localization (fig. S9, B and C). Our nuclear fractionation experiments further confirmed the importance of N terminus for CMT2 nuclear localization (fig. S9D).

### The N terminus of CMT2 regulates protein stability

To investigate the N-terminal function, we generated transgenic *Arabidopsis* plants expressing the full-length AmtriCMT2 as well as an N-terminally truncated AmtriCMT2 without the tandem repeats while retaining the NLS (AmtriCMT2^ΔN^, amino acids 373 to 1183). Unexpectedly, we noted a much higher AmtriCMT2^ΔN^ protein abundance than that of AmtriCMT2, despite similar transcript levels (Fig. 3, D and E). We next asked whether this observed N-terminal function of AmtriCMT2 was shared by *Arabidopsis* CMT2. Instead of truncation, we swapped the N terminus of *Arabidopsis* CMT2 and CMT3 with consideration for the essential role of the NLS (fig. S9). We transformed CMT3 and CMT2^N^-CMT3^ΔN^ into *cmt3* mutant and found that fusion of CMT2 N terminus resulted in much lower CMT3 protein level despite having similar transcript levels (Fig. 3, F and G). Consistently, CMT2 protein level was much higher when the long N terminus was replaced by the shorter CMT3 N terminus (Fig. 3, H and I). These results suggest that the long N terminus regulates CMT2 protein level.

**Fig. 4.**
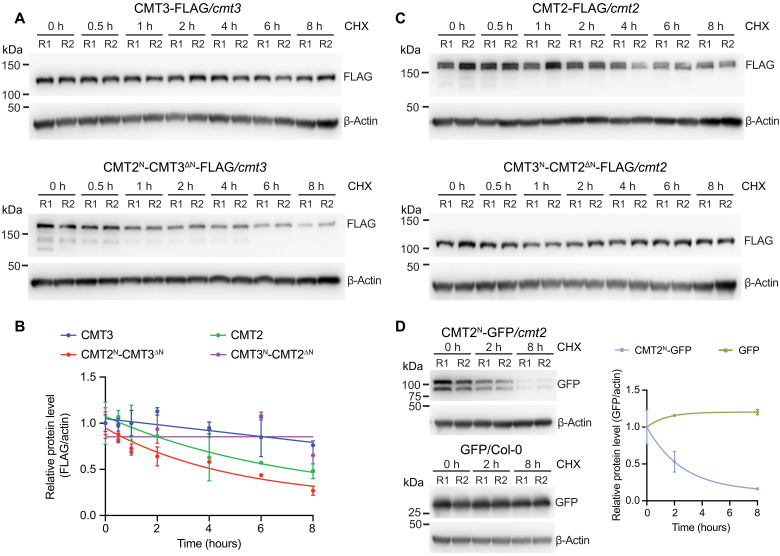
CMT2 N terminus regulates its protein stability. (**A**) Immunoblots showing the levels of CMT3 (top) and CMT2^N^-CMT3^ΔN^ (bottom) after CHX (500 μM) treatment for the indicated time. Seven-day-old seedlings were used. Actin served as a control. R1 and R2 represent two biological replicates. (**B**) Quantification of protein levels normalized to actin. (**C**) Immunoblots showing the levels of CMT2 (top) and CMT3^N^-CMT2^ΔN^ (bottom) after treatment with 500 μM CHX for the indicated time. (**D**) Immunoblots showing levels of CMT2^N^-GFP (top) and GFP only (bottom) after treatment with 500 μM CHX. Quantification of protein levels by GFP signals normalized to actin (right).

We next performed cycloheximide (CHX; a protein synthesis inhibitor) experiment to directly test the CMT2 protein stability. We found that CMT2^N^-CMT3^ΔN^ protein level decreased quickly and was halved after 4 hours of CHX treatment, whereas CMT3 remained relatively stable and decreased at a much slower rate (Fig. 4, A and B). Inversely, CMT2 protein level was halved after 6 hours after CHX treatment, while the CMT3^N^-CMT2^ΔN^ protein remained stable (Fig. 4, B and C). The N terminus alone (CMT2^N^-GFP) was sufficient to affect protein stability in a similar decrease pattern as CMT2^N^-CMT3^ΔN^ (Fig. 4D). Furthermore, a combined treatment of CHX with the 26*S* proteasome inhibitor, MG132, showed that CMT2 protein level was stabilized by the addition of MG132 (fig. S9E). Together, these data showed that the N terminus of CMT2 regulates its protein stability.

### N terminus mediates heat-induced CMT2 degradation

**Fig. 5.**
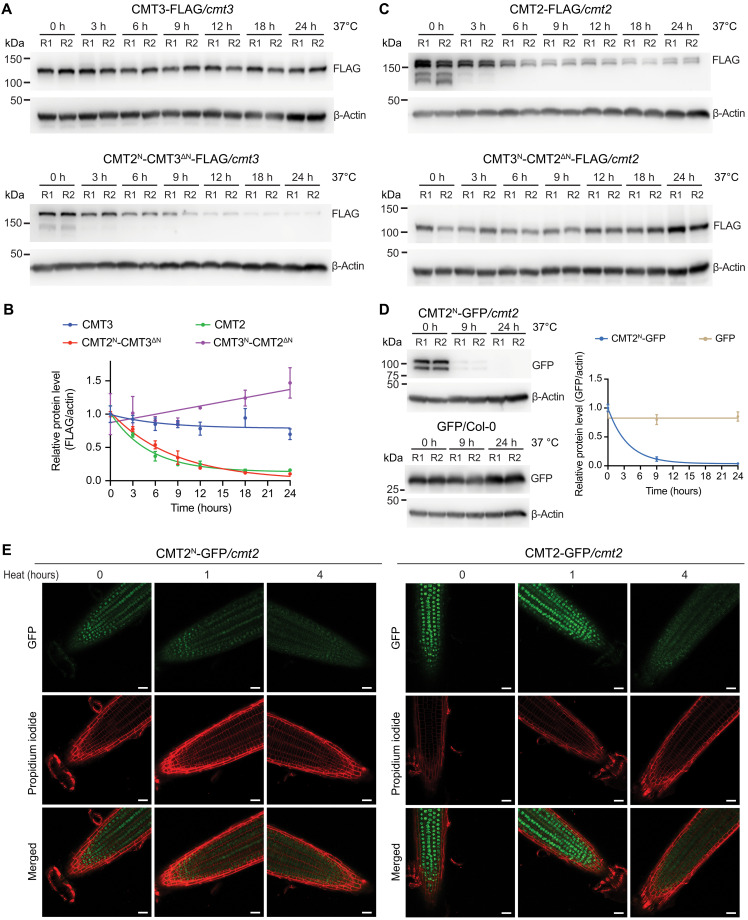
The long N terminus mediates heat-induced CMT2 protein degradation. (**A**) Immunoblots showing the levels of CMT3 (top) and CMT2^N^-CMT3^ΔN^ (bottom) after 37°C heat treatment for the indicated time. Ten-day-old seedlings were used. Actin served as a control. R1 and R2 represent two biological replicates. (**B**) Quantification of protein levels normalized to actin. (**C**) Immunoblots showing the levels of CMT2 (top) and CMT3^N^-CMT2^ΔN^ (bottom) after 37°C heat treatment for the indicated time. (**D**) Immunoblots showing levels of CMT2^N^-GFP (top) and GFP only (bottom) after 37°C heat treatment. Quantification of protein levels by GFP signals normalized to actin (right). (**E**) Confocal images showing the localization and intensity of CMT2^N^-GFP and CMT2-GFP in *Arabidopsis* root tip upon 37°C heat treatment for the indicated time. Scale bars, 25 μm.

We showed previously that CMT2 was degraded upon heat stress and CMT3 remained stable (*17*). To determine whether CMT2 N terminus mediates the heat-induced protein instability, we exposed our transgenic swapped lines to 37°C treatment and found that the fusion of CMT2 N terminus to CMT3 (CMT2^N^-CMT3^ΔN^) demonstrated a much higher sensitivity to heat, where protein level was halved after 6 hours of heat treatment compared to the stable protein level in wild-type CMT3 (Fig. 5, A and B). Conversely, the fusion of N terminus of CMT3 to CMT2 (CMT3^N^-CMT2^ΔN^) greatly increased protein stability under heat stress compared to the fast degradation of wild-type CMT2 (Fig. 5, B and C). Consistently, CMT2^N^-GFP protein abundance decreased and was undetectable after 24 hours of heat treatment (Fig. 5D). Furthermore, we observed reduced nuclear localization of CMT2^N^-GFP upon 4 hours of heat treatment, similar as the full-length CMT2 (Fig. 5E). Together, these results demonstrated that N terminus mediates heat-induced CMT2 degradation.

### Natural variation of CMT2 demonstrated tolerance to environmental stress

**Fig. 6.**
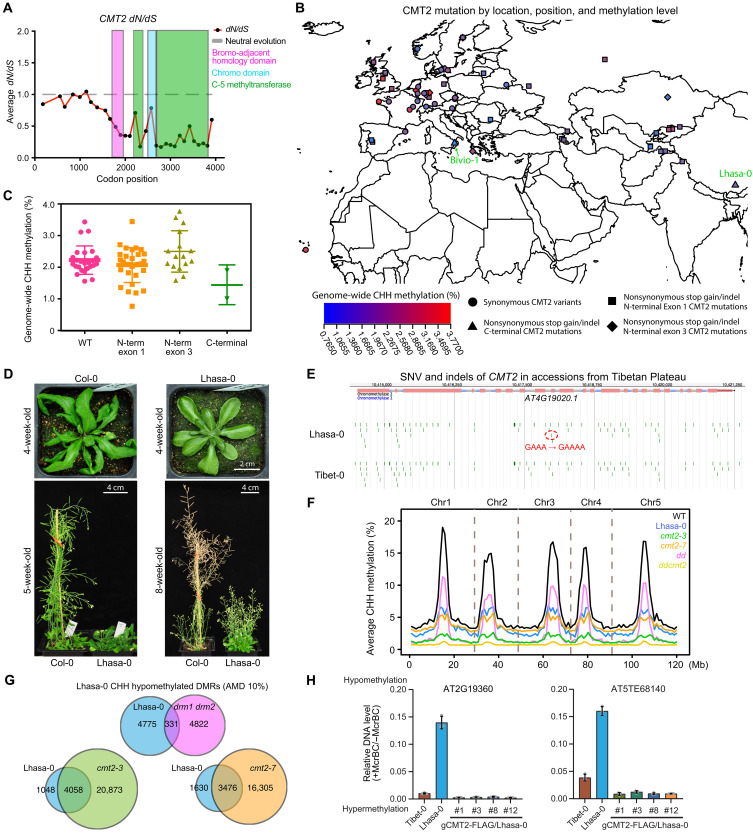
Natural variations of CMT2 in *A. thaliana*. (**A**) *dN*/*dS* plot of *CMT2* gene in close relatives of *A. thaliana*. (**B**) Genome-wide CHH methylation levels of accessions with different mutations in *CMT2* gene. (**C**) Dot plot of CHH methylation in wild-type (WT; *n* = 25), N-terminal [exon 1 (*n* = 31) and exon 3 (*n* = 16) of AT4G19020.1], and C-terminal (*n* = 2) CMT2 mutations. (**D**) Phenotype comparisons between *Arabidopsis* ecotypes Col-0 and Lhasa-0. (**E**) Genome browser view of natural accessions from the Tibetan Plateau, Lhasa-0 and Tibet-0, showing single-nucleotide variant (SNV) with an insertion in the CMT2 exon. (**F**) Average CHH methylation levels across *A. thaliana* chromosomes of Lhasa-0 ecotype in comparison with wild-type Col-0 and mutants of CHH DNA methyltransferases. (**G**) Overlapping of hypomethylated DMRs between Lhasa-0 and mutants of CHH DNA methyltransferases. (**H**) McrBC-qPCR assay showing DNA methylation complementation at two representative loci. The transgenic lines with CMT2 genomic sequence (gCMT2) can rescue lost CHH methylation in Lhasa-0. Tibet-0 was used as a control. AMD, Absolute Methylation Difference.

Many *Arabidopsis* natural accessions with CHH methylation variations have been linked to CMT2 in several genome-wide association studies (*18*, *26*–*30*). Considering the disordered nature of the CMT2 N terminus, we wonder whether the N terminus is more plastic to harbor natural mutations. We examined the CMT2 variations in the 1001 genome project (*31*) and found that the variations were mostly located at the N terminus, especially nonsynonymous mutations (fig. S10A and data S2). Our *dN*/*dS* analysis on the CMT2 coding sequences among close relatives of *A. thaliana* also showed that the N-terminal codons appeared to be evolutionarily neutral (Fig. 6A). Upon reanalysis of BS-seq data from 48 accessions with CMT2 nonsense or frameshift mutations from the 1001 epigenome project (*18*), we found that global CHH methylation level varied with the mutation position in CMT2 and the geographical location of these accessions (Fig. 6B and fig. S10B). Stop-gain mutations in exon 1 and exon 3 are relatively common in nature and appear not to affect genome-wide methylation but may rather increase CHH methylation variability (Fig. 6, B and C). This is consistent with previous studies showing that CMT2 N-terminal variations have relatively weak effects on CHH DNA methylation (*26*, *28*–*30*). This might be caused by the presence of in-frame start codons downstream of these mutations, leading to reinitiation of translation and production of truncated but functional CMT2 protein (fig. S10B) (*32*).

The plasticity of CMT2 N terminus led us to ask whether the C-terminal methyltransferase domain of CMT2 is important for CHH methylation in natural populations. We found one accession, Bivio-1, which contains a 1-bp insertion in the methyltransferase domain coding region and shows lower methylation (Fig. 6B and fig. S10B), suggesting that CMT2 is important for CHH DNA methylation in nature. To gain additional evidence, we analyzed *A. thaliana* accessions from more geographical locations. We found that a natural accession, Lhasa-0, contains a CMT2 mutation in the methyltransferase domain. Lhasa-0 was isolated from the Tibetan plateau (altitude, 4200 m), closely located to Tibet-0 accession (Fig. 6B and fig. S11A) (*33*). Lhasa-0 plants have a broader, more circular leaf structure, dwarf, much more branching, and late flowering compared to Col-0 (Fig. 6D and fig. S11, B and C). We sequenced the genome of Lhasa-0 and that found it contains 1-bp insertion at exon 10 of CMT2, resulting in a frameshift mutation in methyltransferase domain and premature stop codon (Fig. 6E and figs. S10B and S11, D and E). Next, we performed BS-seq and found global CHH methylation reduction in Lhasa-0, to a similar degree of *cmt2* mutants (Fig. 6F). We next called the DMRs using the hcDMR calling program (*34*) and found many hypomethylated CHH DMRs (*n* = 5106) in Lhasa-0 (Fig. 6G). These hypo-DMRs overlapped considerably with *cmt2* but not *drm1drm2* (Fig. 6G), suggesting a link between CMT2 and CHH methylation in Lhasa-0. Furthermore, we found that methylation at the two hypomethylated genes could be rescued by introduction of the genomic fragment of functional *CMT2* transgene into Lhasa-0 (Fig. 6H). These data suggest that CMT2 is responsible for the CHH hypomethylation phenotype of Lhasa-0.

To determine whether mutations in CMT2 could confer any phenotypic advantages or consequences to wild accessions, we performed stress tolerance assays with Col-0, Lhasa-0, and *cmt2* mutants of Col-0. Lhasa-0 showed stronger tolerance to Ultraviolet B (UV-B) stress, while *cmt2-7* also survived in UV-B stress at a slightly higher rate than Col-0 (fig. S11, F and G). In addition, Lhasa-0 and two *cmt2* mutants show resistance to heat stress (fig. S11, H and I), in agreement with a previous report (*29*). Collectively, these data suggest that CMT2 may be involved in plant survival under environmental stresses.

## DISCUSSION

**Fig. 7.**
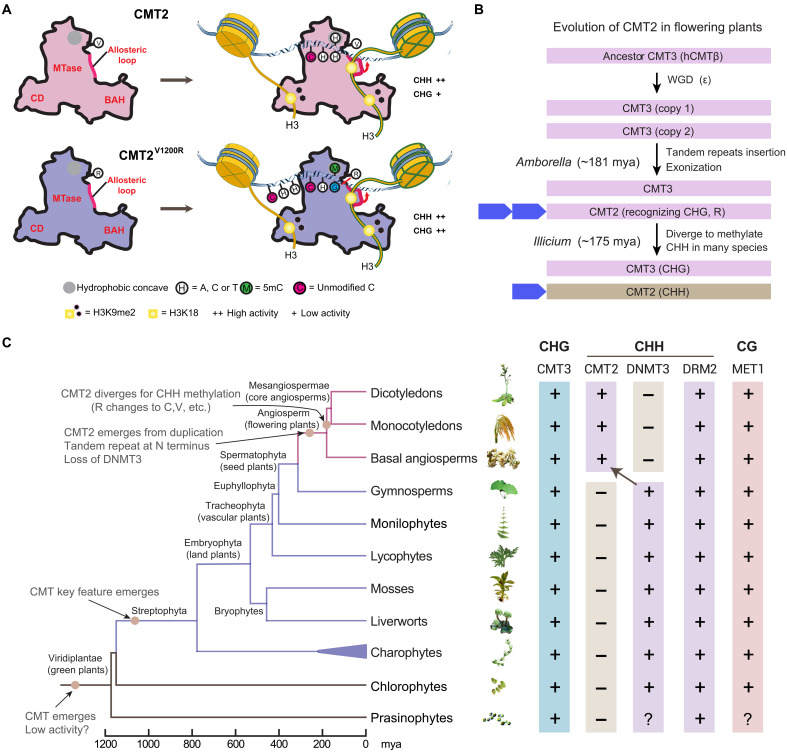
A working model for the evolution of chromomethylases in plants. (**A**) A mechanistic model for the enzymatic regulation and specificity of CMT2 and CMT2^V1200R^ in the chromatin environment. (**B**) Proposed model of CMT2 emergence and evolution in the flowering plants. (**C**) Phylogenetic tree depicting predicted emergence and divergence of chromomethylases in plant (left) and DNA methyltransferases found in the representative plant species (right). MTase, Methyltransferase.

In this study, we revealed the mechanisms diversifying two members of the CMT family, CMT2 and CMT3, for diverse heterochromatic non-CG methylation in plants. According to structural model and biochemical assays, CMT2 and CMT3 share a similar enzymatic mechanism for non-CG methylation, ensuring their overlapped functionalities in non-CG methylation (Fig. 7A). However, an arginine residue that mediates recognition of +2 guanine of CHG via a hydrogen bonding interaction is diversified in angiosperm CMT2, providing an explanation of why CMT3 and CMT2 show distinct preference for CHG and CHH methylation, respectively (*21*). Consistently, a single V1200R mutation of CMT2, which likely restores the hydrogen bonding interaction with the +2 guanine of CHG and enhances the van der Waals or electrostatic contacts with the target-flanking sites of CHG and CHH, gains maintenance activity for both CHG and CHH methylation in *Arabidopsis* (Fig. 7A). During evolution, CMT2 may have evolved from a whole genome duplication event before the emergence of angiosperms about 181 million years ago, as evidenced by the retention of the key arginine CMT3 residue in the CMT2 of basal angiosperm species *A. trichopoda* (Fig. 7B). However, CMT2 is different from CMT3 since its birth, where its N terminus becomes longer, due to a tandem repeat insertion and exonization (Fig. 7B). The long N terminus is disordered, undergoes neutral selection during evolution, and makes the protein relatively unstable. Nevertheless, the N terminus contains several conserved tandem RRS motifs, although the number and position vary in flowering plants. The arginine variation together with the long N terminus drives the evolution of CMT2 for CHH methylation preference.

It is worth noting that recent studies have indicated that DNMT3s, which are homologous to mammalian DNMT3A and DNMT3B, also play a role in CHH methylation maintenance in heterochromatic regions in some plant species such as the moss *Physcomitrella patens* (*23*, *35*). Intriguingly, DNMT3s are widely present in the plant kingdom but have become extinct in angiosperms (Fig. 7C). In this context, the evolutionary emergence of CMT2 presumably provides a similar, possibly even more robust, mechanism in replacing the loss of DNMT3-mediated CHH methylation and maintaining the non-CG methylation at heterochromatin (Fig. 7C). The functional context behind the evolutionary transition between CMT2 and DNMT3s awaits further investigation.

CMT2 appears to be more plastic and could offer a great target for evolution, where it contains much more variations in nature or is even lost in certain angiosperm species. In maize, CMT2 is lost, and the CMT3 (ZMET2) has both CHG and CHH methylation activity, yet whether this could be a reason for the high TE abundance and activity remains to be investigated (*9*, *36*). It was previously reported that TEs in *A. thaliana* natural accessions varied greatly, corresponding to differences in gene expression and DNA methylation in these accessions, which could provide a source for genetic diversity in the population (*37*). We found an abundance of natural variations in CMT2, especially in the N terminus. We also identified a new *A. thaliana* accession Lhasa-0 carrying a CMT2 mutation leading to global DNA hypomethylation. The globally decreased CHH methylation in Lhasa-0, Bivio-1, and a recently reported Kyoto, which contains an 8-bp deletion at exon 3 close to the methyltransferase domain, supports the importance of CMT2 on CHH methylation in natural conditions (*27*). As Lhasa-0 is isolated from Tibetan Plateau, a high-altitude environment with harsh conditions such as strong sunshine and UV-B radiation, the mutation in CMT2 could possibly confer some tolerance to environmental stresses (*33*, *38*). Thus, over evolutionary time, mutations in CMT2 could potentially alleviate repression on TEs which could then increase genetic diversity. Consistent with this idea, we found that another species, *Arabis alpina*, a close relative of *A. thaliana* from high-altitude alpines, also contains a CMT2 mutation with the loss of the essential BAH domain (fig. S12). TEs, especially retrotransposons, have also been expanded, accompanied with altered DNA methylation in *A. alpina* (*39*). The transcriptional activity of TEs also seems to increase along with an elevational gradient in *Arabidopsis arenosa* (*40*). Previously, we found that UV-B also triggered DNA hypomethylation via the interaction between UVR8 and DRM2 (*41*). Whether CMT2 is also involved in UV-B–suppressed DNA methylation requires further investigation.

CMT2 stability is sensitive to heat stress, mainly conferred by its long and disordered N terminus. Although the N terminus varies in different angiosperm species, many contain the conserved RRS motifs, indicating that these motifs may have yet undiscovered functions. Degradation of CMT2 under high temperature could impede its function for CHH methylation maintenance at heterochromatic TEs, thereby alleviating some TEs that were normally suppressed under normal conditions. Over the course of evolution, some derepressed TEs could potentially jump in the genome and generate various genetic mutations for natural selection, which could be especially beneficial for species survival under stress. The variation of TE transposition in *A. thaliana* wild accessions is related to climate change, where the increase in annual temperature correlates with an increase of *AtCopia87* (*ONSEN*) copy number (*42*). As another support, natural CMT2 variation has been associated with genome-wide methylation changes and temperature seasonality (*29*). During the Earth history and in a large period, the average global temperature is much higher (up to more than 10°C) than that of today (*43*). Thus, we can infer that during the hothouse era, the instability of CMT2 together with the TEs may have helped shape the plant evolution on earth.

## MATERIALS AND METHODS

### Plant materials, plasmid construction, and transformation

*A. thaliana* ecotypes Col-0, Tibet-0, and Lhasa-0 (gifted from Y. Zhong) were used in this study. The Col-0 ecotype was used as the background for all mutant and transgenic plants, except for experiments in the Lhasa-0 ecotype. Mutant lines used in this study were *cmt3* (*cmt3-11*, SALK_148381), *cmt2* (*cmt2-3* SALK_012874 and *cmt2-7*, WiscDsLox7E02), *cc* (*cmt2-7 cmt3-11*, *cmt2-7* is WiscDsLox7E02), and *ddcc* (*drm1-2 drm2-2 cmt3-11 cmt2-7*). All seedlings and plants used in this study were grown in long-day conditions (16/8-hour light/dark cycle) at 22°C in a Conviron chamber. *N. benthamiana* plants were grown directly on soil and under long-day conditions.

Genomic fragments of *Arabidopsis* CMT2 or CMT3 were used to construct plasmids for transgenic lines. Using genomic DNA extracted by the Cetyltrimethylammonium bromide (CTAB) method as the template, genomic fragments including the native promoter (CMT2, 1.4 kb; CMT3, 1.6 kb), 5′ untranslated region, exons, and introns were amplified by polymerase chain reaction (PCR) and then ligated to linearized vector by in-fusion cloning method (Vazyme, C115). For FLAG-tagged lines, the vector pCAMBIA-FLAG-FAST containing a 3× FLAG tag at the C terminus of the genomic fragment and a selection marker pOLE1:OLE1-RFP, which is specifically expressed in dry seeds, was used (*44*). For GFP-tagged lines, the vector pCAMBIA1302 containing an *mgfp5* marker gene was used. For the expression of CMT2 and CMT3 of other plant species, e.g., *A. trichopoda* (from Botany Garden and Greenhouses of University of Wisconsin-Madison), *C. braunii* (from Kobe University), and *M. polymorpha* (collected from Madison, WI), the protein-coding sequence was amplified from cDNA and fused after the UBQ10 promoter and ligated to linearized pCAMBIA-FLAG-FAST vector.

Transgenic lines used in this study include *gCMT3-FLAG* and *gCMT3-GFP*, domain truncation and swapping generated in *cmt3* and *cc* backgrounds; *gCMT2-FLAG*, *gCMT2^V1200R^-FLAG*, and *gCMT2-GFP*, domain truncation and swapping generated in *cc*, *cmt2*, *cmt3*, and *ddcc* backgrounds; CMT2 and CMT3 of other species were generated in respective *Arabidopsis cmt2* and *cmt3* backgrounds. Transgenic plants were generated via a floral-dip method and screened with a hand-held fluorescent lamp (Luyor-3415RG) for OLE1-RFP expression in seeds or by hygromycin selection. For CMT2 complementation in Lhasa-0 background, genomic CMT2 fragment from Col-0 was transformed into Lhasa-0 plants using similar methods as above.

### Immunoblotting

Frozen plant samples were ground to powder with a tissue lyser (Scientzbio). Proteins were extracted by boiling sample in 5% SDS at 95°C for 10 min. Proteins were separated by SDS–polyacrylamide gel electrophoresis and transferred to nitrocellulose membranes. Membranes were blocked with blocking buffer [5% nonfat milk in tris-buffered saline with Tween 20 (TBST)] for 1 hour at room temperature and then incubated with primary antibodies [diluted in 3% bovine serum albumin (BSA) in TBST] overnight at 4^°^C. For unconjugated primary antibodies, membranes were incubated with horseradish peroxidase (HRP)–conjugated secondary antibodies in blocking buffer for 1 hour at room temperature. Membranes were washed with TBST for 5 min, three times, before imaging. Enhanced chemiluminescence substrate was mixed and added to the membrane before imaging, and chemiluminescence images were taken with ImageQuant LAS 4000 (GE Healthcare) or Odyssey XF (LI-COR). The primary antibodies used were anti-FLAG-HRP (Sigma-Aldrich, catalog no. A8592, RRID:AB_439702), anti-GFP (Roche, catalog no. 11814460001, RRID:AB_390913), anti–β-actin (Proteintech, catalog no. 60008-1-Ig, RRID:AB_2289225), anti–α-tubulin (Novus, catalog no. NB100-690, RRID:AB_521686), and anti-histoneH3 (Abcam, catalog no. ab1791, RRID:AB_302613).

For nuclear extraction, frozen seedlings (~1.5 g per sample) were gently ground into powder with liquid nitrogen using a mortar and pestle. Nuclei were isolated with a protocol modified by Chen *et al.* (*45*). Briefly, ground powder was mixed in nuclei extraction buffer (tris, Ficoll 400, Dextran T40, sucrose, and MgCl_2_) with dithiothreitol (DTT), phenylmethylsulfonyl fluoride, and cOmplete protease cocktail added right before use and then filtered through a 70-μm cell strainer followed by a 40-μm cell strainer. Filtered samples were adjusted with Triton X-100 to 0.5% (v/v) and incubated on ice for 15 min. A small portion of sample was collected (total protein extract) before centrifugation at 1500*g* for 5 min at 4°C. After centrifugation, the supernatant (cytoplasmic fraction) was collected, while the pellet (nuclear fraction) was washed three to four times by resuspending the pellet gently in nuclei extraction buffer with 0.1% Triton-X and then centrifuged at 1800*g* for 5 min at 4°C. The nuclear fraction was prepared by resuspending the pellet in 150 μl of nuclei extraction buffer. Each fraction was mixed with 5× loading buffer and boiled at 95°C for 10 min for immunoblotting.

### CHX and MG132 treatment

CHX stock (25 mM; Sigma-Aldrich, 01810) was prepared in Milli-Q water. For treatment, 7-day-old seedlings were submerged in six-well plates containing 500 μM CHX in half-strength Murashige and Skoog (½ Murashige and Skoog) liquid media. Samples were collected at indicated time points by drying briefly on Kimwipes and immediately frozen in liquid nitrogen to prevent further degradation. MG132 treatment was performed as described previously (*46*). Briefly, 7-day-old seedlings were submerged in six-well plates containing 500 μM CHX with the addition of 50 μM MG132 (Sigma-Aldrich, C2211) or dimethyl sulfoxide in ½ MS liquid media. Samples were then collected as described above.

### Heat treatment

For heat treatment, 10-day-old seedlings were treated directly on plates in Conviron growth chamber at 37°C. Samples were collected at indicated time points and immediately frozen in liquid nitrogen to prevent further degradation. For confocal microscopy, 6-day-old seedlings were treated at 37°C at indicated time points and imaged immediately after heat treatment.

### Confocal microscopy

To determine transient protein expression, Agrobacterium-infiltrated *N. benthamiana* leaf sections were imaged with a Leica Stellaris 5 confocal microscope. To determine in vivo protein localization, root tips of 7-day-old *Arabidopsis* transgenic seedlings expressing CMT2 or CMT3 fused with GFP were imaged with a Leica Stellaris 5 confocal microscope. To determine protein expression and localization after heat treatment, 6-day-old seedlings were imaged immediately after heat treatment with a Leica SP8 Upright confocal microscope.

### Quantitative real-time PCR analysis

For quantitative reverse transcription PCR (RT-qPCR), plant total RNA was extracted using the Ambion PureLink RNA Mini Kit (Invitrogen). First strand cDNA was then synthesized from 1 μg of the extracted total RNA using random hexamer, anchored oligodT_18_VN, murine ribonuclease inhibitor, and ProtoScript II [New England Biolabs (NEB)] reverse transcriptase. For McrBC assay, plant genomic DNA was extracted with the CTAB method. An equal amount of genomic DNA was then digested with the methylation-dependent restriction endonuclease McrBC (NEB) for 8 hours at 37°C. The quantitative real-time PCR was performed in triplicates using iTaq Universal SYBR Green Supermix and ran with the Bio-Rad CFX Opus 96 Real-Time PCR System (Bio-Rad). The gene expression levels in RT-qPCR were normalized against wild-type control and internal control *ACT7*. The relative methylation levels of McrBC-qPCR were normalized to uncut control.

### Protein expression and purification

Expression and purification of wild-type and mutant CMT2 follow a similar protocol as previously described for CMT3 (*21*). In short, each of the CMT2-encoding sequences was cloned into a modified PRSFDuet-1 vector (Novagen), fused with an N-terminal His6-SUMO tag. The expression plasmids were then transformed into *Escherichia coli* BL21 (DE3) cells for protein expression. The His6-SUMO-CMT2 proteins were purified through nickel affinity chromatography, followed by removal of His6-SUMO tag via ubiquitin-like-specific protease 1–mediated cleavage. The tag-free CMT2 proteins were further purified through ion-exchange chromatography on a Heparin column (GE Healthcare) and size-exclusion chromatography on a 16/600 Superdex 200 pg column (GE Healthcare). The purified protein sample was stored in buffer containing 20 mM tris-HCl (pH 8.0), 100 mM NaCl, 5 mM DTT, and 5% glycerol in a −80°C freezer before use.

### In vitro methylation assay

In vitro methylation assay was performed as described previously (*21*). In essence, the H3_1-17_K9me2 and H3_1-32_K9me2 peptides, each followed by a C-terminal tyrosine, were chemically synthesized and verified by mass spectroscopy. Each 20-μl reaction mixture contains 0.5 μM wild-type or mutant CMT2, 2 μM histone peptide, 2 μM synthesized DNA duplexes (hmCHG: 5′-AATATATXTGXAGXTGAATXAGXAGXTGTAATTTAA-3′, annealed with unmethylated, complimentary strand; X = 5mC; CHG: 5′-TGCTGCTGCTGCTGCTGCTGCTGCTGCTGCTGCTGCTGCTGCTGC-3′, annealed with unmethylated, complimentary strand; CHH: 5′-TACTACTACTACTACTACTACTACTACTACTACTACTACTACTAC-3′, annealed with complimentary strand), 0.56 μM *S*-adenosyl-l-[methyl-^3^H] methionine with a specific activity of 18 Ci/mmol (PerkinElmer), 1.96 μM nonradioactive S-adenosyl methionine (SAM), 50 mM tris-HCl (pH 8.0), 0.05% β-mercaptoethanol, 5% glycerol, and BSA (200 μg/ml). The reaction lasted 30 min at 37°C before being quenched by addition of 5 μl of 10 mM cold SAM. Subsequently, 8 μl of each mixture was loaded onto Hybond N nylon membrane (GE Healthcare), which was left being dried out at room temperature. The membrane was then washed with 0.2 M ammonium bicarbonate (pH 8.2) three times, 5 min each, deionized water for 5 min once, and 95% ethanol for 5 min once. After air drying, the membrane carrying each sample was transferred into a vial containing 4-ml scintillation buffer (Thermo Fisher Scientific). The tritium scintillation was measured and recorded by a Beckman LS6500 counter. Each of the reactions was repeated three times.

### BS-seq and data analysis

For whole genome BS-seq, seeds of Col-0, *cmt3-11*, pUBQ10:MpCMT3-FLAG/*cmt3-11*, pUBQ10:CbCMT3-FLAG/*cmt3-11*, *cc*, gCMT2-FLAG, and corresponding V1200R mutation in *cc* background were planted on ½ MS medium for 10 days. Genomic DNA was then extracted from whole seedlings using the CTAB method. The genomic DNA was fragmented to a mean size of 100 to 300 bp by sonication using a Covaris S220 focused-ultrasonicator (Covaris), followed by end-repair, 3′-end adenylation, and methylated adaptor ligation using an Illumina TruSeq DNA kit (Illumina). Then, bisulfite conversion was performed using a Zymo EZ DNA Methylation-Lightning kit (Zymo Research). The bisulfite-converted, adaptor-ligated DNA was enriched by PCR for 12 cycles using the KAPA HiFi HotStart Uracil+ Kit (KAPA Biosystems), purified with Agencourt beads (Agilent), and quantified by a Qubit HS dsDNA kit (Life Technologies). The integrity of the sequencing library was tested by Agilent 2100. The libraries related to CMT3 were sequenced by a 50-bp paired-end method on a NextSeq2000 platform at Delaware Biotechnology Institute in University of Delaware (Newark, DE, USA), and the libraries related to V1200R mutation were sequenced by a 50-bp single-end method on a HiSeq4000 platform at NUcore sequencing center in Northwestern University (Chicago, IL, USA). Sequencing reads were trimmed using FASTP and aligned to the *A. thaliana* TAIR10 genome using Bisulfite Sequence MAPping program (BSMAP) version 2.9 allowing for 4% mismatches, trimming anything with a quality score of 33 or less, and removing any reads with more than five Ns (*47*, *48*). Methylation at every cytosine was determined by using BSMAP’s methratio.py script, processing only unique reads and removing duplicates. DMRs were identified using methylKit with the following cutoffs for each sequence context: 40% difference for CG, 20% for CHG, and 10% difference for CHH DMRs and a *P* value < 0.01 (*49*). Overlapping DMRs were identified using BEDTools intersectBED (*50*). TE metaplots were created using deepTools computeMatrix using BSMAP methylation file and a list of all TEs from TAIR10 (*51*). Whole genome methylation metaplots were created using the BSMAP methylation file and custom python scripts. Boxplots were created using custom R scripts.

### RNA sequencing and analysis

For RNA sequencing, seeds of Col-0, *cc*, and two lines of each indicated gCMT2-FLAG and corresponding V1200R mutation were planted on ½ MS medium containing 1% sucrose for 10 days in long-day growth conditions (16-hour light:8-hour dark) at 22°C. Total RNA was extracted using the PureLink RNA Mini Kit and treated with deoxyribonuclease I. Library was constructed using a TruSeq RNA Library Preparation Kit (Illumina); mRNA was purified using RNA purification beads and fragmented by sonication using a Covaris S220 focused-ultrasonicator. One microgram of total RNA was reverse-transcribed into cDNA with ProtoScript II. End-repair, 3′-end adenylation, ligation of adaptors and PCR amplification for 13 cycles were then performed. Reads were filtered using fastp and then aligned them to the TAIR10 genome using HISAT2 (v.2.0.0-beta) (*52*). Transcripts were assembled using StringTie (*53*). The quantification of gene expression and the identification of DEGs were performed with DESeq2 (*54*). The heatmap was made using Heatmapper (http://heatmapper.ca/expression/). Differentially up-regulated and down-regulated genes were analyzed using GO Term Enrichment tool on TAIR/PANTHER, and fold enrichment was calculated by dividing the number of DEGs by the expected number of DEGs for a GO term (*55*).

### Other sequence analysis

For Assay for Transposase-Accessible Chromatin with sequencing (ATAC-seq) analysis, raw data were downloaded from GSE85203 and aligned to the TAIR10 genome using Bowtie2 with the following parameter: -v 2 -m 3 (*56*). PCR duplicates were then removed using SAMtools (*57*). The uniquely mapped reads were kept and used for chromatin immunoprecipitation (ChIP) peaks calling with MACS2 (*58*). DMRs were overlapped with ChIP peaks using BEDTools intersectBed (*50*). Coverage bed graphs were generated using bamCompare for histone modifications normalizing to H3 and bamCoverage for ATAC-seq normalized with reads per kilobase per million mapped reads (RPKM) values (*51*). The level of each modification within the DMRs was calculated using a custom python script, and boxplots were generated using a custom R script.

### Sequence from multiple species and analysis

Sequences of CMT2 and CMT3 from multiple plant species were retrieved by Basic Local Alignment Search Tool (BLAST) using Ensembl Plants (plants.ensembl.org), National Center for Biotechnology Information (NCBI), 1000 Plant transcriptomes initiative (OneKP) (db.cngb.org/onekp), PLAZA Gymnosperms (bioinformatics.psb.ugent.be/plaza/versions/gymno-plaza), FernBase (fernbase.org), or PhycoCosm (phycocosm.jgi.doe.gov). The sequences are listed in data S1. The disorder scores were determined using the VSL2 algorithm of PONDR (*59*). The natural variations of CMT2 were from Araport11/TAIR JBrowse (https://jbrowse.arabidopsis.org/).

### Lhasa-0 sequence analysis

Paired-end whole genome DNA sequencing libraries [used for single-nucleotide variant (SNV) calling] were prepared by Starbio using in-house library preparation protocols. Paired-end BS-seq libraries were prepared. One hundred fifty–base pair paired-end sequencing was performed for both library types on an Illumina Hi-Seq 2500 instrument. Read alignment statistics can be found in table S1. To generate pseudoreference genomes for Tibet-0 and Lhasa-0, raw fastqs were croptrimmed for phred score of ≥30 using Trimmomatic and aligned to the TAIR10 genome (Col-0 ecotype) using Bowtie2 using the–local alignment setting, 1-bp mismatch in 22-bp seeds (*56*, *60*). SNVs and indels for Tibet-0 and Lhasa-0 were called against TAIR10 using the SAMtools mpileup function to compute likelihood of the data given each possible genotype and bcftools applied the priors to call variant sites. ANNOtate VARiation (ANNOVAR) was used to do a gene-based functional annotation of genetic variants (*61*). The variant called format (.vcf) files were used to produce a pseudoreference genome via the pseudoRef R-package (https://github.com/yangjl/pseudoRef).

For Tibet-0 and Lhasa-0 BS-seq analyses, data were initially aligned to the assembled pseudoreference genome via BSMAP version 2.9 (*48*). Reads were filtered for <5 N and quality trimmed (Phred score ≥ 30), and alignment was allowed proceed with up to 8% read mismatch (default). Methylation tracks were created by calling cytosine methylation in all contexts via the methratio.py script removing duplicated reads and including zeros. Whole genome plots were produced by averaging DNA methylation in each context (CG, CHG, and CHH) in 1-Mb bins via a custom python script (https://github.com/Sandman2127/Whole_Genome_DNA_Methylation_Plotter/blob/master/WG_methylation_plotter.py) and plotted using the R packages ggplot2 and reshape2 (*62*, *63*). After alignment via BSMAP, 100-bp DMRs were called against 54 wild-type high-confidence *Arabidopsis* libraries via hcDMR caller, accepting only unique mappings and using the -Steve filter (*34*, *48*). Metaplots of DNA methylation were produced using deepTools compute Matrix and profiler functions at DMRs (*51*).

### 1001 Epigenome BS-seq data reanalysis

Data from the 1001 epigenomes project were reanalyzed using the above parameters in BSMAP with previously built pseudoreference genomes (http://1001genomes.org/data/GMI-MPI/releases/v3.1/pseudogenomes/fasta/) (*64*). CHH methylation averages were averaged genome-wide using an awk script and plotted via the R-package rworldmap (https://CRAN.R-project.org/package=rworldmap).

### *dN*/*dS* analysis

Analysis of evolutionary constraint on the CMT2 gene by *dN*/*dS* was performed by generating a multiple sequence alignment for the CDS of CMT2 with *A. thaliana*, *Arabidopsis lyrata*, *Begonia grandis*, *Brassica rapa*, *C. rubella*, and *Brachypodium distachyon* using ClustalW from Mega7 (*65*). Selection at each codon was estimated using the HyPhy (*66*). The data were interpreted according to the principles found previously (*67*).

### Statistical analysis

The two-tailed Student’s *t* tests were performed to compare distributions between different groups. For all tests, a *P* value lower than 0.05 was statistically significant.

## Supplementary Materials

### The PDF file includes:

Figs. S1 to S12 Tables S1 and S2 Legends for data S1 to S3 References

## References

[1] A. L. Mattei, N. Bailly, A. Meissner, DNA methylation: A historical perspective. Trends Genet. 38, 676–707 (2022). 35504755 10.1016/j.tig.2022.03.010

[2] R. J. Schmitz, Z. A. Lewis, M. G. Goll, DNA methylation: Shared and divergent features across eukaryotes. Trends Genet. 35, 818–827 (2019). 31399242 10.1016/j.tig.2019.07.007 PMC6825889

[3] M. V. C. Greenberg, D. Bourc’his, The diverse roles of DNA methylation in mammalian development and disease. Nat. Rev. Mol. Cell Biol. 20, 590–607 (2019). 31399642 10.1038/s41580-019-0159-6

[4] L. He, H. Huang, M. Bradai, C. Zhao, Y. You, J. Ma, L. Zhao, R. Lozano-Duran, J. K. Zhu, DNA methylation-free *Arabidopsis* reveals crucial roles of DNA methylation in regulating gene expression and development. Nat. Commun. 13, 1335 (2022). 35288562 10.1038/s41467-022-28940-2 PMC8921224

[5] S. M. Leichter, J. Du, X. Zhong, Structure and mechanism of plant DNA methyltransferases. Adv. Exp. Med. Biol. 1389, 137–157 (2022). 36350509 10.1007/978-3-031-11454-0_6 PMC10112988

[6] H. Zhang, Z. Lang, J. K. Zhu, Dynamics and function of DNA methylation in plants. Nat. Rev. Mol. Cell Biol. 19, 489–506 (2018). 29784956 10.1038/s41580-018-0016-z

[7] S. Henikoff, L. Comai, A DNA methyltransferase homolog with a chromodomain exists in multiple polymorphic forms in Arabidopsis. Genetics 149, 307–318 (1998). 9584105 10.1093/genetics/149.1.307 PMC1460135

[8] A. M. Lindroth, X. Cao, J. P. Jackson, D. Zilberman, C. M. McCallum, S. Henikoff, S. E. Jacobsen, Requirement of CHROMOMETHYLASE3 for maintenance of CpXpG methylation. Science 292, 2077–2080 (2001). 11349138 10.1126/science.1059745

[9] A. J. Bewick, C. E. Niederhuth, L. Ji, N. A. Rohr, P. T. Griffin, J. Leebens-Mack, R. J. Schmitz, The evolution of CHROMOMETHYLASES and gene body DNA methylation in plants. Genome Biol. 18, 65 (2017). 28457232 10.1186/s13059-017-1195-1 PMC5410703

[10] R. Ren, H. Wang, C. Guo, N. Zhang, L. Zeng, Y. Chen, H. Ma, J. Qi, Widespread whole genome duplications contribute to genome complexity and species diversity in angiosperms. Mol. Plant 11, 414–428 (2018). 29317285 10.1016/j.molp.2018.01.002

[11] E. Kuzmin, J. S. Taylor, C. Boone, Retention of duplicated genes in evolution. Trends Genet. 38, 59–72 (2022). 34294428 10.1016/j.tig.2021.06.016 PMC8678172

[12] J. Du, X. Zhong, Y. V. Bernatavichute, H. Stroud, S. Feng, E. Caro, A. A. Vashisht, J. Terragni, H. G. Chin, A. Tu, J. Hetzel, J. A. Wohlschlegel, S. Pradhan, D. J. Patel, S. E. Jacobsen, Dual binding of chromomethylase domains to H3K9me2-containing nucleosomes directs DNA methylation in plants. Cell 151, 167–180 (2012). 23021223 10.1016/j.cell.2012.07.034 PMC3471781

[13] A. Zemach, M. Y. Kim, P. H. Hsieh, D. Coleman-Derr, L. Eshed-Williams, K. Thao, S. L. Harmer, D. Zilberman, The Arabidopsis nucleosome remodeler DDM1 allows DNA methyltransferases to access H1-containing heterochromatin. Cell 153, 193–205 (2013). 23540698 10.1016/j.cell.2013.02.033 PMC4035305

[14] H. Stroud, T. Do, J. Du, X. Zhong, S. Feng, L. Johnson, D. J. Patel, S. E. Jacobsen, Non-CG methylation patterns shape the epigenetic landscape in *Arabidopsis*. Nat. struct. Mol. Biol. 21, 64–72 (2014). 24336224 10.1038/nsmb.2735 PMC4103798

[15] J. M. Wendte, Y. Zhang, L. Ji, X. Shi, R. R. Hazarika, Y. Shahryary, F. Johannes, R. J. Schmitz, Epimutations are associated with CHROMOMETHYLASE 3-induced de novo DNA methylation. elife 8, e47891 (2019). 31356150 10.7554/eLife.47891 PMC6663294

[16] A. J. Bewick, L. Ji, C. E. Niederhuth, E. M. Willing, B. T. Hofmeister, X. Shi, L. Wang, Z. Lu, N. A. Rohr, B. Hartwig, C. Kiefer, R. B. Deal, J. Schmutz, J. Grimwood, H. Stroud, S. E. Jacobsen, K. Schneeberger, X. Zhang, R. J. Schmitz, On the origin and evolutionary consequences of gene body DNA methylation. Proc. Natl. Acad. Sci. U.S.A. 113, 9111–9116 (2016). 27457936 10.1073/pnas.1604666113 PMC4987809

[17] K. Nozawa, J. Chen, J. Jiang, S. M. Leichter, M. Yamada, T. Suzuki, F. Liu, H. Ito, X. Zhong, DNA methyltransferase CHROMOMETHYLASE3 prevents ONSEN transposon silencing under heat stress. PLOS Genet. 17, e1009710 (2021). 34411103 10.1371/journal.pgen.1009710 PMC8376061

[18] T. Kawakatsu, S.-S. C. Huang, F. Jupe, E. Sasaki, R. J. Schmitz, M. A. Urich, R. Castanon, J. R. Nery, C. Barragan, Y. He, H. Chen, M. Dubin, C.-R. Lee, C. Wang, F. Bemm, C. Becker, R. O’Neil, R. C. O’Malley, D. X. Quarless; 101 Genomes Consortium, N. J. Schork, D. Weigel, M. Nordborg, J. R. Ecker, Epigenomic diversity in a global collection of Arabidopsis thaliana accessions. Cell 166, 492–505 (2016). 27419873 10.1016/j.cell.2016.06.044 PMC5172462

[19] L. He, C. Zhao, Q. Zhang, G. Zinta, D. Wang, R. Lozano-Duran, J. K. Zhu, Pathway conversion enables a double-lock mechanism to maintain DNA methylation and genome stability. Proc. Natl. Acad. Sci. U.S.A. 118, e2107320118 (2021). 34453006 10.1073/pnas.2107320118 PMC8536323

[20] C. I. Stoddard, S. Feng, M. G. Campbell, W. Liu, H. Wang, X. Zhong, Y. Bernatavichute, Y. Cheng, S. E. Jacobsen, G. J. Narlikar, A nucleosome bridging mechanism for activation of a maintenance DNA methyltransferase. Mol. Cell 73, 73–83.e6 (2019). 30415948 10.1016/j.molcel.2018.10.006 PMC6407616

[21] J. Fang, J. Jiang, S. M. Leichter, J. Liu, M. Biswal, N. Khudaverdyan, X. Zhong, J. Song, Mechanistic basis for maintenance of CHG DNA methylation in plants. Nat. Commun. 13, 3877 (2022). 35790763 10.1038/s41467-022-31627-3 PMC9256654

[22] C. Noy-Malka, R. Yaari, R. Itzhaki, A. Mosquna, N. Auerbach Gershovitz, A. Katz, N. Ohad, A single CMT methyltransferase homolog is involved in CHG DNA methylation and development of Physcomitrella patens. Plant Mol. Biol. 84, 719–735 (2014). 24370935 10.1007/s11103-013-0165-6

[23] R. Yaari, A. Katz, K. Domb, K. D. Harris, A. Zemach, N. Ohad, RdDM-independent de novo and heterochromatin DNA methylation by plant CMT and DNMT3 orthologs. Nat. Commun. 10, 1613 (2019). 30962443 10.1038/s41467-019-09496-0 PMC6453930

[24] C. Cheng, Y. Tarutani, A. Miyao, T. Ito, M. Yamazaki, H. Sakai, E. Fukai, H. Hirochika, Loss of function mutations in the rice chromomethylase OsCMT3a cause a burst of transposition. Plant J. 83, 1069–1081 (2015). 26243209 10.1111/tpj.12952

[25] Amborella Genome Project, The *Amborella* genome and the evolution of flowering plants. Science 342, 1241089 (2013). 24357323 10.1126/science.1241089

[26] M. J. Dubin, P. Zhang, D. Meng, M. S. Remigereau, E. J. Osborne, F. Paolo Casale, P. Drewe, A. Kahles, G. Jean, B. Vilhjalmsson, J. Jagoda, S. Irez, V. Voronin, Q. Song, Q. Long, G. Ratsch, O. Stegle, R. M. Clark, M. Nordborg, DNA methylation in Arabidopsis has a genetic basis and shows evidence of local adaptation. eLife 4, e05255 (2015). 25939354 10.7554/eLife.05255 PMC4413256

[27] K. Nozawa, S. Masuda, H. Saze, Y. Ikeda, T. Suzuki, H. Takagi, K. Tanaka, N. Ohama, X. Niu, A. Kato, H. Ito, Epigenetic regulation of ecotype-specific expression of the heat-activated transposon ONSEN. Front. Plant Sci. 13, 899105 (2022). 35923888 10.3389/fpls.2022.899105 PMC9340270

[28] E. Sasaki, T. Kawakatsu, J. R. Ecker, M. Nordborg, Common alleles of CMT2 and NRPE1 are major determinants of CHH methylation variation in Arabidopsis thaliana. PLOS Genet. 15, e1008492 (2019). 31887137 10.1371/journal.pgen.1008492 PMC6953882

[29] X. Shen, J. De Jonge, S. K. G. Forsberg, M. E. Pettersson, Z. Sheng, L. Hennig, O. Carlborg, Natural CMT2 variation is associated with genome-wide methylation changes and temperature seasonality. PLOS Genet 10, e1004842 (2014). 25503602 10.1371/journal.pgen.1004842 PMC4263395

[30] E. Sasaki, J. Gunis, I. Reichardt-Gomez, V. Nizhynska, M. Nordborg, Conditional GWAS of non-CG transposon methylation in *Arabidopsis thaliana* reveals major polymorphisms in five genes. PLOS Genet. 18, e1010345 (2022). 36084135 10.1371/journal.pgen.1010345 PMC9491579

[31] The 1001 Genomes Consortium, 1,135 genomes reveal the global pattern of Polymorphism in Arabidopsis thaliana. Cell 166, 481–491 (2016). 27293186 10.1016/j.cell.2016.05.063 PMC4949382

[32] S. Cohen, L. Kramarski, S. Levi, N. Deshe, O. Ben David, E. Arbely, Nonsense mutation-dependent reinitiation of translation in mammalian cells. Nucleic Acids Res. 47, 6330–6338 (2019). 31045216 10.1093/nar/gkz319 PMC6614817

[33] L. Zeng, Z. Gu, M. Xu, N. Zhao, W. Zhu, T. Yonezawa, T. Liu, L. Qiong, T. Tersing, L. Xu, Y. Zhang, R. Xu, N. Sun, Y. Huang, J. Lei, L. Zhang, F. Xie, F. Zhang, H. Gu, Y. Geng, M. Hasegawa, Z. Yang, M. J. C. Crabbe, F. Chen, Y. Zhong, Discovery of a high-altitude ecotype and ancient lineage of Arabidopsis thaliana from Tibet. Sci. Bull. 62, 1628–1630 (2017). 10.1016/j.scib.2017.10.007 36659379

[34] Y. Zhang, C. J. Harris, Q. Liu, W. Liu, I. Ausin, Y. Long, L. Xiao, L. Feng, X. Chen, Y. Xie, X. Chen, L. Zhan, S. Feng, J. J. Li, H. Wang, J. Zhai, S. E. Jacobsen, Large-scale comparative epigenomics reveals hierarchical regulation of non-CG methylation in *Arabidopsis*. Proc. Natl. Acad. Sci. U.S.A 115, E1069–E1074 (2018). 29339507 10.1073/pnas.1716300115 PMC5798360

[35] G. Malik, M. Dangwal, S. Kapoor, M. Kapoor, Role of DNA methylation in growth and differentiation in Physcomitrella patens and characterization of cytosine DNA methyltransferases. FEBS J. 279, 4081–4094 (2012). 22943564 10.1111/febs.12002

[36] Q. Gouil, D. C. Baulcombe, DNA methylation signatures of the plant chromomethyltransferases. PLOS Genet. 12, e1006526 (2016). 27997534 10.1371/journal.pgen.1006526 PMC5221884

[37] T. Stuart, S. R. Eichten, J. Cahn, Y. V. Karpievitch, J. O. Borevitz, R. Lister, Population scale mapping of transposable element diversity reveals links to gene regulation and epigenomic variation. eLife 5, e20777 (2016). 27911260 10.7554/eLife.20777 PMC5167521

[38] M. Zhang, J. Zhao, W. Y. Li, S. Q. Wen, H. L. Huang, J. Dong, B. Liu, G. Zhang, H. B. Wang, Y. T. Shen, H. L. Jin, Increased photosystem II translation efficiency as an important photoprotective mechanism in an *Arabidopsis thaliana* (Tibet-0) adapted to high light environments. Environ. Exp. Bot. 183, 104350 (2021).

[39] E.-M. Willing, V. Rawat, T. Mandakova, F. Maumus, G. V. James, K. J. V. Nordstrom, C. Becker, N. Warthmann, C. Chica, B. Szarzynska, M. Zytnicki, M. C. Albani, C. Kiefer, S. Bergonzi, L. Castaings, J. L. Mateos, M. C. Berns, N. Bujdoso, T. Piofczyk, L. de Lorenzo, C. Barrero-Sicilia, I. Mateos, M. Piednoel, J. Hagmann, R. Chen-Min-Tao, R. Iglesias-Fernandez, S. C. Schuster, C. Alonso-Blanco, F. Roudier, P. Carbonero, J. Paz-Ares, S. J. Davis, A. Pecinka, H. Quesneville, V. Colot, M. A. Lysak, D. Weigel, G. Coupland, K. Schneeberger, Genome expansion of *Arabis alpina* linked with retrotransposition and reduced symmetric DNA methylation. Nat. Plants 1, 14023 (2015). 27246759 10.1038/nplants.2014.23

[40] G. Wos, R. R. Choudhury, F. Kolar, C. Parisod, Transcriptional activity of transposable elements along an elevational gradient in Arabidopsis arenosa. Mob. DNA 12, 7 (2021). 33639991 10.1186/s13100-021-00236-0 PMC7916287

[41] J. Jiang, J. Liu, D. Sanders, S. Qian, W. Ren, J. Song, F. Liu, X. Zhong, UVR8 interacts with de novo DNA methyltransferase and suppresses DNA methylation in *Arabidopsis*. Nat. Plants 7, 184–197 (2021). 33495557 10.1038/s41477-020-00843-4 PMC7889724

[42] L. Quadrana, A. Bortolini Silveira, G. F. Mayhew, C. LeBlanc, R. A. Martienssen, J. A. Jeddeloh, V. Colot, The Arabidopsis thaliana mobilome and its impact at the species level. eLife 5, e15716 (2016). 27258693 10.7554/eLife.15716 PMC4917339

[43] T. Westerhold, N. Marwan, A. J. Drury, D. Liebrand, C. Agnini, E. Anagnostou, J. S. K. Barnet, S. M. Bohaty, D. De Vleeschouwer, F. Florindo, T. Frederichs, D. A. Hodell, A. E. Holbourn, D. Kroon, V. Lauretano, K. Littler, L. J. Lourens, M. Lyle, H. Palike, U. Rohl, J. Tian, R. H. Wilkens, P. A. Wilson, J. C. Zachos, An astronomically dated record of Earth’s climate and its predictability over the last 66 million years. Science 369, 1383–1387 (2020). 32913105 10.1126/science.aba6853

[44] T. L. Shimada, T. Shimada, I. Hara-Nishimura, A rapid and non-destructive screenable marker, FAST, for identifying transformed seeds of Arabidopsis thaliana. Plant J. 61, 519–528 (2010). 19891705 10.1111/j.1365-313X.2009.04060.x

[45] X. Chen, L. Lu, K. S. Mayer, M. Scalf, S. Qian, A. Lomax, L. M. Smith, X. Zhong, POWERDRESS interacts with HISTONE DEACETYLASE 9 to promote aging in *Arabidopsis*. elife 5, e17214 (2016). 27873573 10.7554/eLife.17214 PMC5119886

[46] J. Chen, J. Liu, J. Jiang, S. Qian, J. Song, R. Kabara, I. Delo, G. Serino, F. Liu, Z. Hua, X. Zhong, F-box protein CFK1 interacts with and degrades de novo DNA methyltransferase in Arabidopsis. New Phytol. 229, 3303–3317 (2021). 33216996 10.1111/nph.17103 PMC7902366

[47] S. Chen, Y. Zhou, Y. Chen, J. Gu, fastp: An ultra-fast all-in-one FASTQ preprocessor. Bioinformatics 34, i884–i890 (2018). 30423086 10.1093/bioinformatics/bty560 PMC6129281

[48] Y. Xi, W. Li, BSMAP: Whole genome bisulfite sequence MAPping program. BMC Bioinformatics 10, 232 (2009). 19635165 10.1186/1471-2105-10-232 PMC2724425

[49] A. Akalin, M. Kormaksson, S. Li, F. E. Garrett-Bakelman, M. E. Figueroa, A. Melnick, C. E. Mason, methylKit: A comprehensive R package for the analysis of genome-wide DNA methylation profiles. Genome Biol. 13, R87 (2012). 23034086 10.1186/gb-2012-13-10-r87 PMC3491415

[50] A. R. Quinlan, I. M. Hall, BEDTools: A flexible suite of utilities for comparing genomic features. Bioinformatics 26, 841–842 (2010). 20110278 10.1093/bioinformatics/btq033 PMC2832824

[51] F. Ramirez, D. P. Ryan, B. Gruning, V. Bhardwaj, F. Kilpert, A. S. Richter, S. Heyne, F. Dundar, T. Manke, deepTools2: A next generation web server for deep-sequencing data analysis. Nucleic Acids Res. 44, W160–W165 (2016). 27079975 10.1093/nar/gkw257 PMC4987876

[52] D. Kim, J. M. Paggi, C. Park, C. Bennett, S. L. Salzberg, Graph-based genome alignment and genotyping with HISAT2 and HISAT-genotype. Nat. Biotechnol. 37, 907–915 (2019). 31375807 10.1038/s41587-019-0201-4 PMC7605509

[53] M. Pertea, G. M. Pertea, C. M. Antonescu, T. C. Chang, J. T. Mendell, S. L. Salzberg, StringTie enables improved reconstruction of a transcriptome from RNA-seq reads. Nat. Biotechnol. 33, 290–295 (2015). 25690850 10.1038/nbt.3122 PMC4643835

[54] M. I. Love, W. Huber, S. Anders, Moderated estimation of fold change and dispersion for RNA-seq data with DESeq2. Genome Biol. 15, 550 (2014). 25516281 10.1186/s13059-014-0550-8 PMC4302049

[55] H. Mi, A. Muruganujan, X. Huang, D. Ebert, C. Mills, X. Guo, P. D. Thomas, Protocol update for large-scale genome and gene function analysis with the PANTHER classification system (v.14.0). Nat. Protoc. 14, 703–721 (2019). 30804569 10.1038/s41596-019-0128-8 PMC6519457

[56] B. Langmead, S. L. Salzberg, Fast gapped-read alignment with Bowtie 2. Nat. Methods 9, 357–359 (2012). 22388286 10.1038/nmeth.1923 PMC3322381

[57] H. Li, B. Handsaker, A. Wysoker, T. Fennell, J. Ruan, N. Homer, G. Marth, G. Abecasis, R. Durbin; 1000 Genome Project Data Processing Subgroup, The Sequence Alignment/Map format and SAMtools. Bioinformatics 25, 2078–2079 (2009). 19505943 10.1093/bioinformatics/btp352 PMC2723002

[58] Y. Zhang, T. Liu, C. A. Meyer, J. Eeckhoute, D. S. Johnson, B. E. Bernstein, C. Nusbaum, R. M. Myers, M. Brown, W. Li, X. S. Liu, Model-based analysis of ChIP-Seq (MACS). Genome Biol. 9, R137 (2008). 18798982 10.1186/gb-2008-9-9-r137 PMC2592715

[59] B. Xue, R. L. Dunbrack, R. W. Williams, A. K. Dunker, V. N. Uversky, PONDR-FIT: a meta-predictor of intrinsically disordered amino acids. Biochim. Biophys. Acta 1804, 996–1010 (2010). 20100603 10.1016/j.bbapap.2010.01.011 PMC2882806

[60] A. M. Bolger, M. Lohse, B. Usadel, Trimmomatic: A flexible trimmer for Illumina sequence data. Bioinformatics 30, 2114–2120 (2014). 24695404 10.1093/bioinformatics/btu170 PMC4103590

[61] K. Wang, M. Li, H. Hakonarson, ANNOVAR: Functional annotation of genetic variants from high-throughput sequencing data. Nucleic Acids Res. 38, e164 (2010). 20601685 10.1093/nar/gkq603 PMC2938201

[62] H. Wickham, “Use R!” in *Ggplot2: Elegant Graphics for Data Analysis* (Springer, 1st ed., 2009).

[63] H. Wickham, Reshaping data with the reshape package. J. Stat. Softw. 21, 1–20 (2007).

[64] A. J. Bewick, R. J. Schmitz, Epigenetics in the wild. eLife 4, e07808 (2015). 25939361 10.7554/eLife.07808 PMC4417933

[65] S. Kumar, G. Stecher, K. Tamura, MEGA7: Molecular evolutionary genetics analysis version 7.0 for bigger datasets. Mol. Biol. Evol. 33, 1870–1874 (2016). 27004904 10.1093/molbev/msw054 PMC8210823

[66] S. L. K. Pond, S. D. W. Frost, S. V. Muse, HyPhy: Hypothesis testing using phylogenies. Bioinformatics 21, 676–679 (2005). 15509596 10.1093/bioinformatics/bti079

[67] S. Kryazhimskiy, J. B. Plotkin, The population genetics of dN/dS. PLOS Genet. 4, e1000304 (2008). 19081788 10.1371/journal.pgen.1000304 PMC2596312

[68] Y. Liu, T. Tian, K. Zhang, Q. You, H. Yan, N. Zhao, X. Yi, W. Xu, Z. Su, PCSD: A plant chromatin state database. Nucleic Acids Res. 46, D1157–D1167 (2018). 29040761 10.1093/nar/gkx919 PMC5753246

