# Supplementary material for: Substrate specificity and protein stability drive the divergence of plant-specific DNA methyltransferases
Source: Sci Adv. 2024 Nov 6;10(45):eadr2222. doi: 10.1126/sciadv.adr2222 (PMC11540031; doi:10.1126/sciadv.adr2222)
Supplement: Supplementary file 1 — Figs. S1 to S12 Tables S1 and S2 Legends for data S1 to S3 References [file sciadv.adr2222_sm.pdf]

Science Advances
AAAS

Supplementary Materials for

Substrate specificity and protein stability drive the divergence of plant-specific DNA methyltransferases

Jianjun Jiang *et al.*

Corresponding author: Xuehua Zhong, xuehuazhong@wustl.edu; Jikui Song, jikui.song@ucr.edu

Sci. Adv. **10**, eadr2222 (2024)
DOI: 10.1126/sciadv.adr2222

The PDF file includes:

Figs. S1 to S12
Tables S1 and S2
Legends for data S1 to S3
References

Other Supplementary Material for this manuscript includes the following:

Data S1 to S3

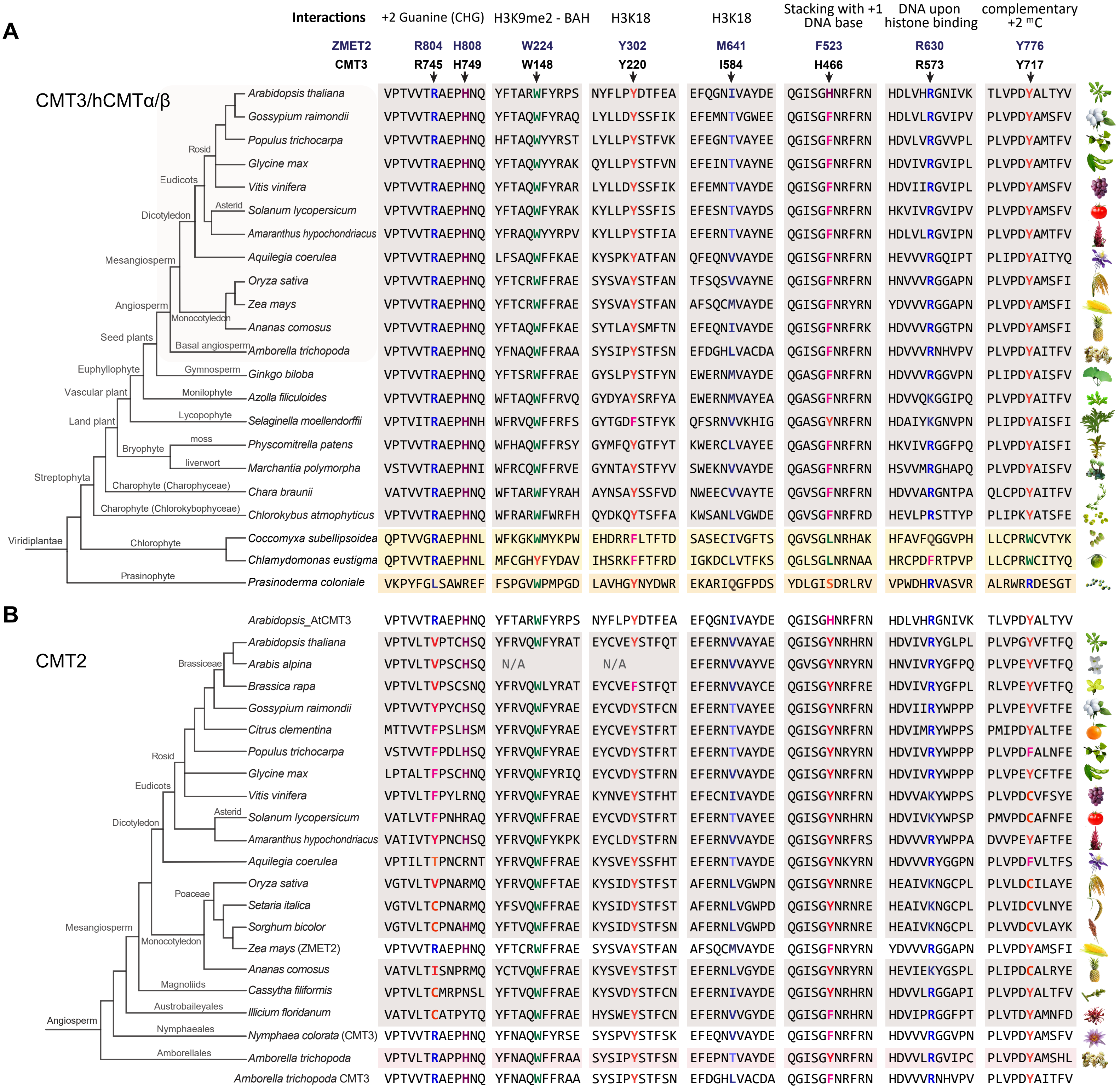

Figure S1. Key residues for CMT-type DNA methyltransferases in phylogenetic tree.
(**A** and **B**) Phylogenetic trees depicting the conservation of key residues of CMT3 (**A**) and CMT2 (**B**) in plants. CMT3 is present in green plants (*Viridiplantae*), while CMT2 is only present in flowering plants (angiosperms) and lost in some species during evolution.

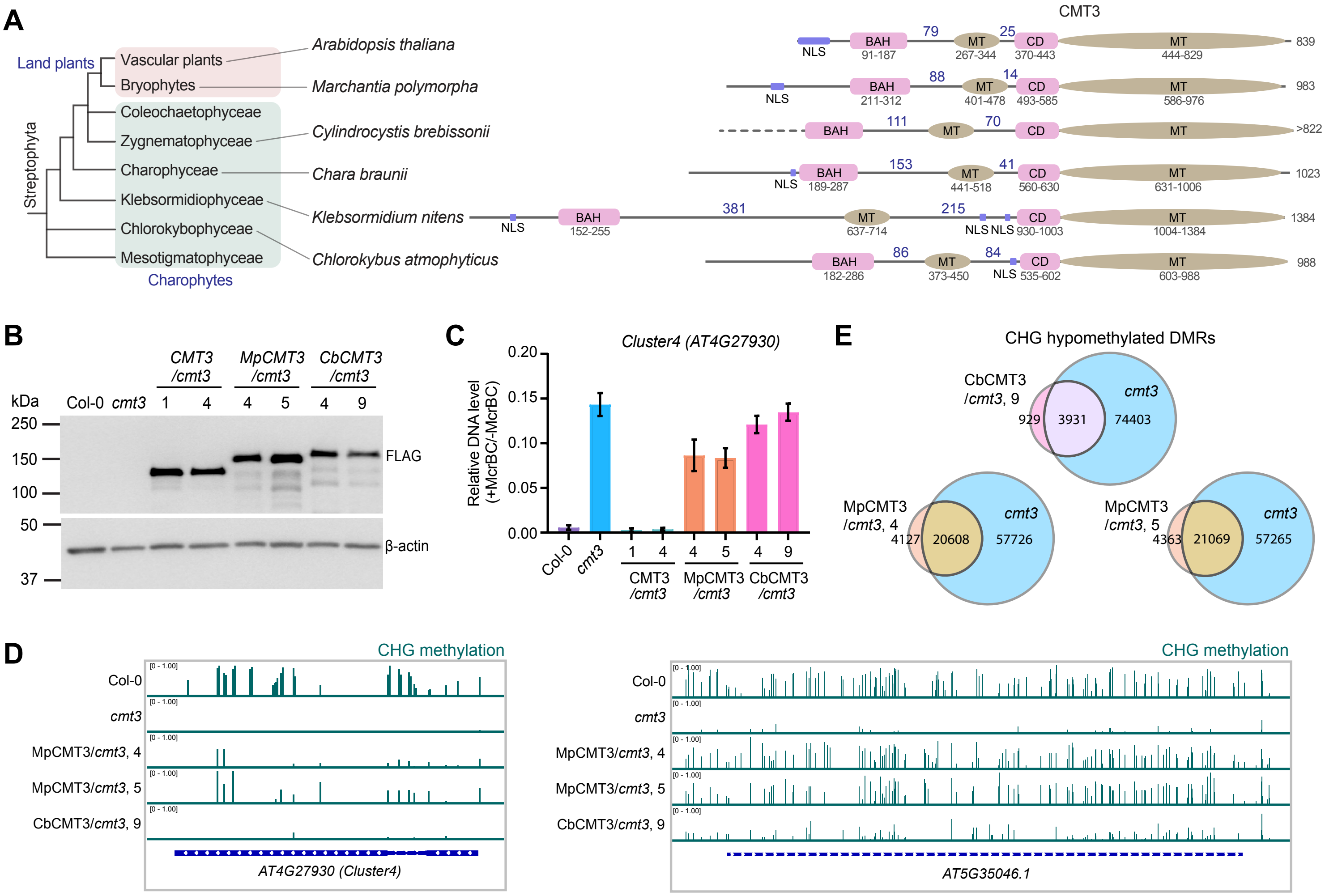

Figure S2. The CHG methylation activity of CMT3 is conserved across plant evolution.
(**A**) Phylogenetic tree showing Charophytes and land plant species that share similar domain layouts in their CMT3 proteins. Nuclear localization signal (NLS) colored in violet. BAH: Bromo-Adjacent Homology domain; MT: methyltransferase domain; CD: chromo domain. Numbers above lines depict the number of amino acids between each domain. (**B**) Immunoblots showing protein levels of *C. braunii* and *M. polymorpha* CMT3 transformed into *Arabidopsis cmt3* mutant background under the control of *UBQ10* promoter. (**C**) DNA methylation level over a CMT3 targeted locus, *Cluster4*, by *C. braunii* CMT3 (CbCMT3) and *M. polymorpha* CMT3 (MpCMT3) compared to wildtype (Col-0) and transgenic CMT3 measured by McrBC-qPCR assay. (**D**) Genome browser view of CHG methylation levels of two loci from bisulfite-sequencing data. (**E**) Venn diagrams showing overlapping of CHG DMRs identified by comparing indicated genotypes with Col-0 in GSE39901.

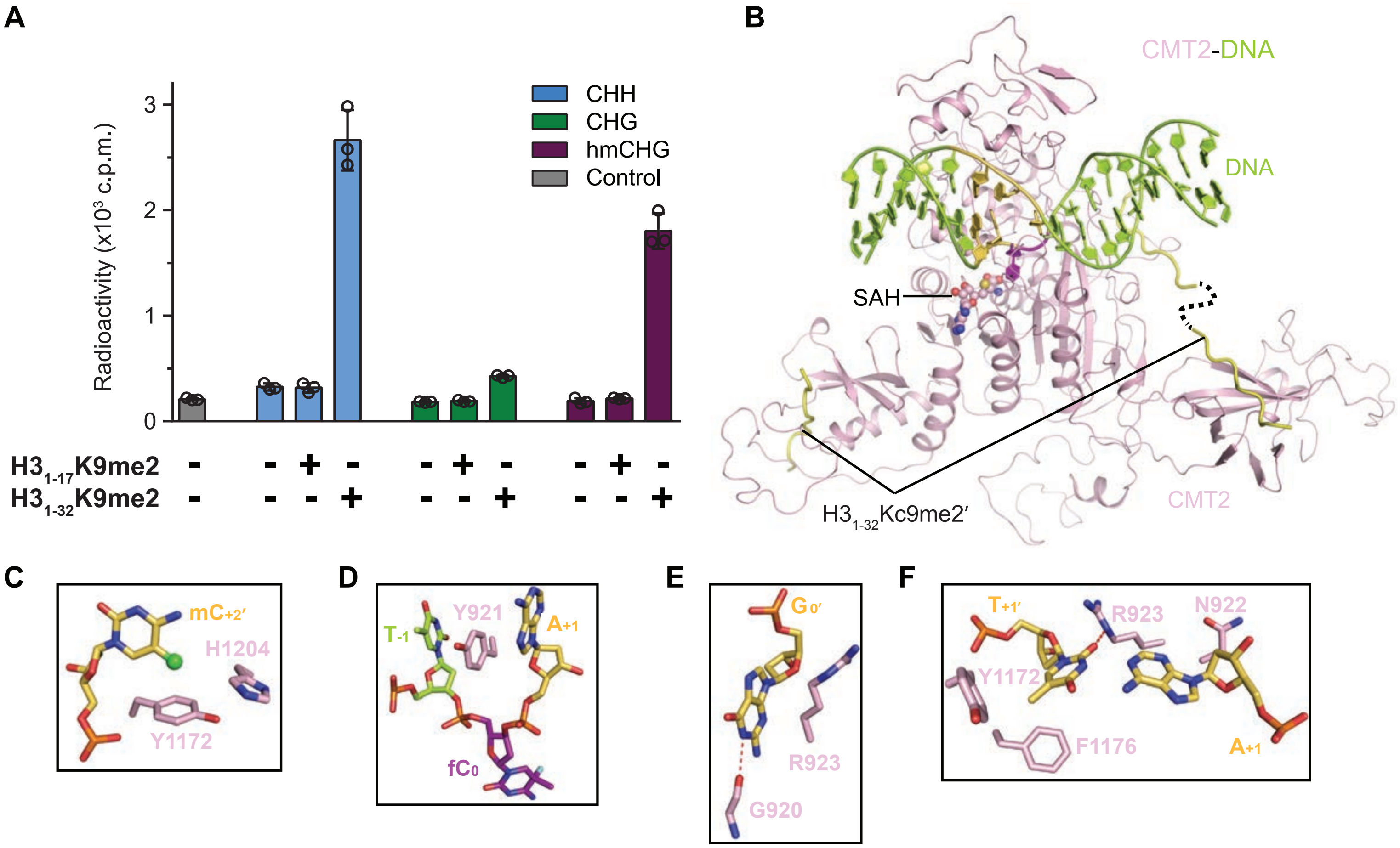

Figure S3. Predicted model of CMT2 interaction with DNA in CHG context.
(**A**) *In vitro* DNA methylation assay of CMT2 on DNA duplex containing multiple CHH, CHG, or hmCHG sites, in the presence or absence of H3K9me2 peptides. Control, no substrate present. (**B**) Structural prediction of CMT2 interacting with DNA based on crystal structure of ZMET2 in Fang *et al.* (*21*). (**C**) Close-up predictions of CMT2-DNA interactions at the +2-flanking $mC_{+2'}$. (**D**) Of CMT2-DNA interactions at the DNA cavity vacated by base flipping of fC_0 on the target strand. (**E**) Of CMT2-DNA interactions at the orphan $G_{0'}$ site on the template strand. (**F**) Of CMT2-DNA interactions at the +1-flanking $A_{+1}{\cdot}T_{+1'}$ pair.

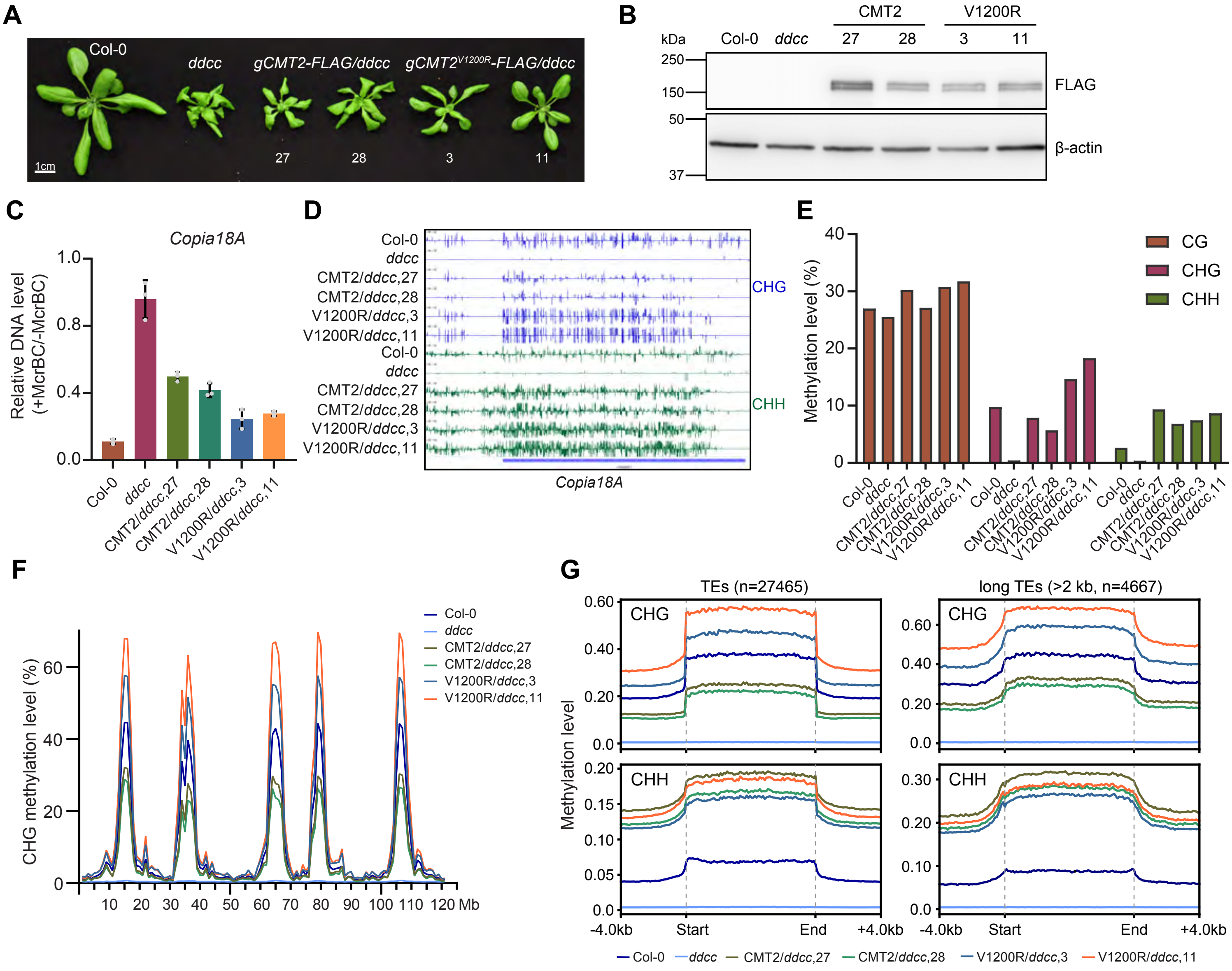

Figure S4. Effects of V1200R mutation on CHG methylation in *cmt3* and *ddcc* mutant backgrounds.
(**A**) Phenotypes of 3-week-old transgenic CMT2 and CMT2^{V1200R} plants in *drm1drm2cmt2cmt3* (*ddcc*) quadruple mutant background. (**B**) Immunoblots showing CMT2 and V1200R protein levels in *ddcc* background with actin as a loading control. (**C**) DNA methylation levels of CMT2 and V1200R over two CMT2-targeted TEs in *ddcc* measured by McrBC-qPCR assay and normalized with no-enzyme controls. (**D**) Genome browser view of CHG and CHH methylation levels of CMT2 and V1200R in *ddcc* background at *Copia18A* site. (**E**) Proportion of DNA methylation in CG, CHG, and CHH contexts of transgenic CMT2 and V1200R in *ddcc* background. (**F**) Metaplots for the CHG methylation across the *Arabidopsis* genome in CMT2 and V1200R in *ddcc* background. The peaks represent the centromeres and near centromere regions of the five chromosomes. (**G**) Metaplots of CHG and CHH methylation over all TEs (n=27465) in *Arabidopsis* genome in CMT2 and V1200R in *ddcc* background and over long TEs (> 2kb, n=4667).

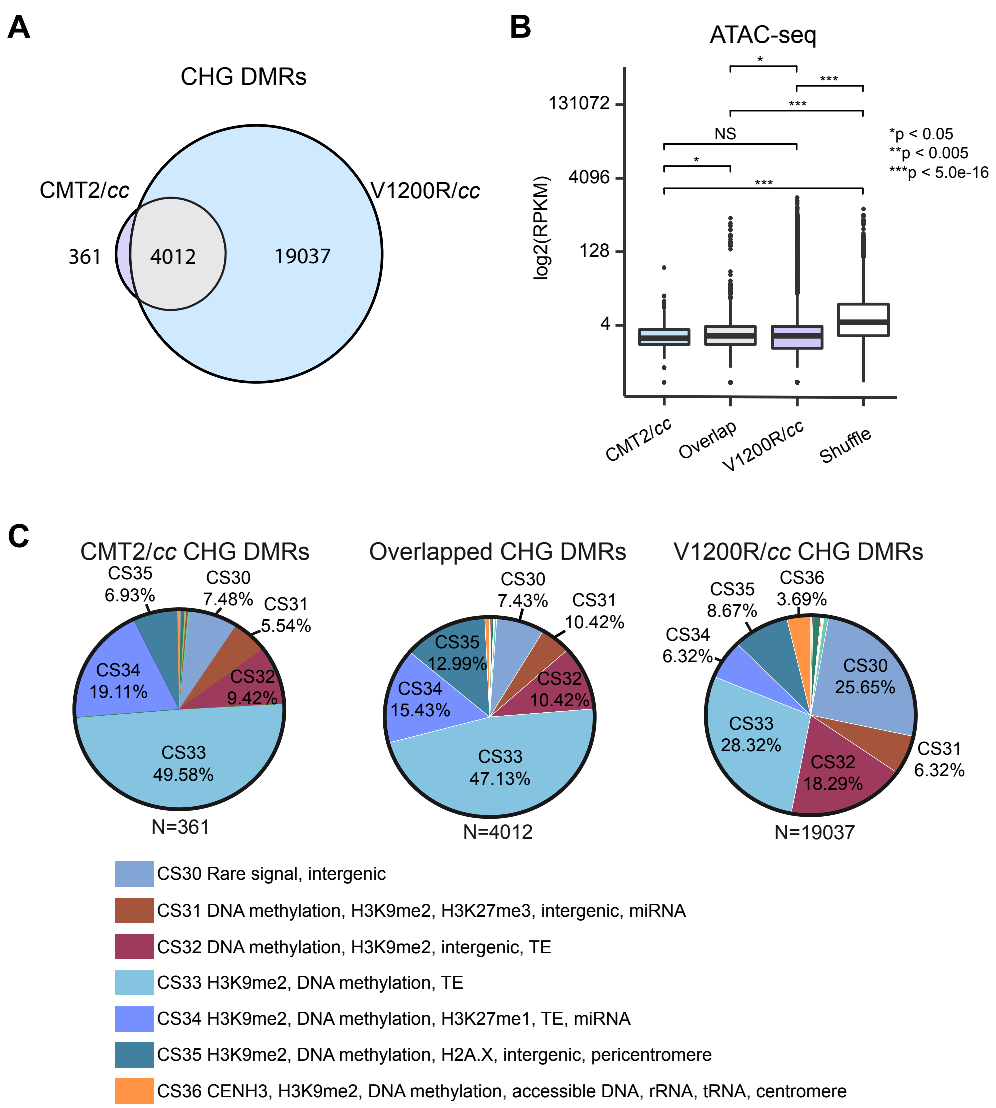

Figure S5. The chromatin environment of CMT2^{V1200R} methylated regions.
(**A**) Venn diagram depicting the overlap between CMT2 and V1200R CHG DMRs determined by the overlap in DMRs of two biological replicates. Only DMR demonstrated 100% overlap between two datasets is considered as an overlap. (**B**) Boxplots showing the levels of ATAC-seq in Col-0 at CMT2 and V1200R CHG DMRs. * p < 0.05, ** p < 0.005, ***p < 5.0e-16 by Wilcoxon test. NS, not significant. (**C**) Pie charts showing the proportion of CHG DMRs that falls into chromatin states defined by Liu *et al*. (*68*).

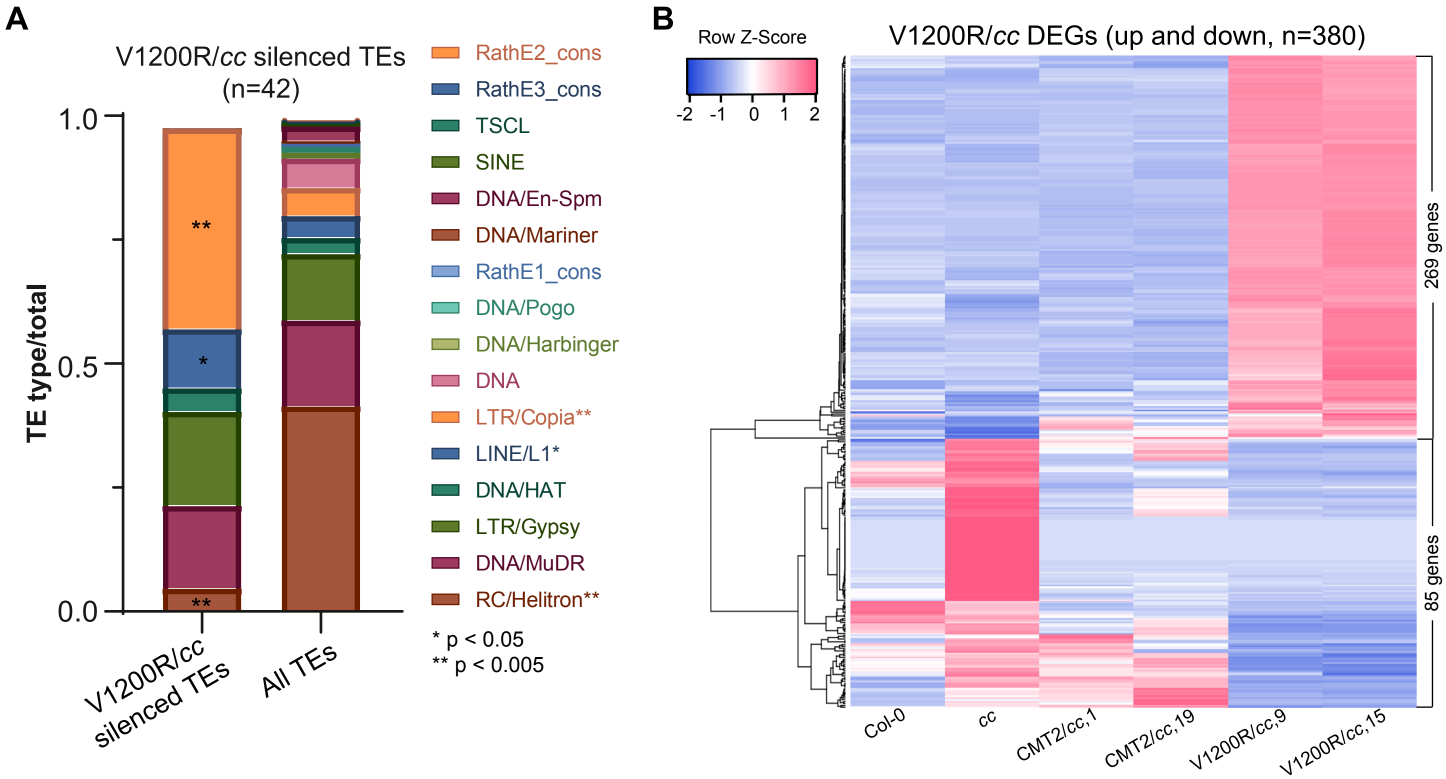

Figure S6. TEs and genes regulated by CMT2^{V1200R}.
(**A**) Enrichment of TE types in TEs expressed in CMT2 but silenced in Col-0 and V1200R (shown in Figure 2A). *p < 0.05, **p < 0.005 by Fisher's Exact test. (**B**) Heat map showing the expression level of 380 shared DEGs (both up and down) between two biological replicates listed in Fig. 2D.

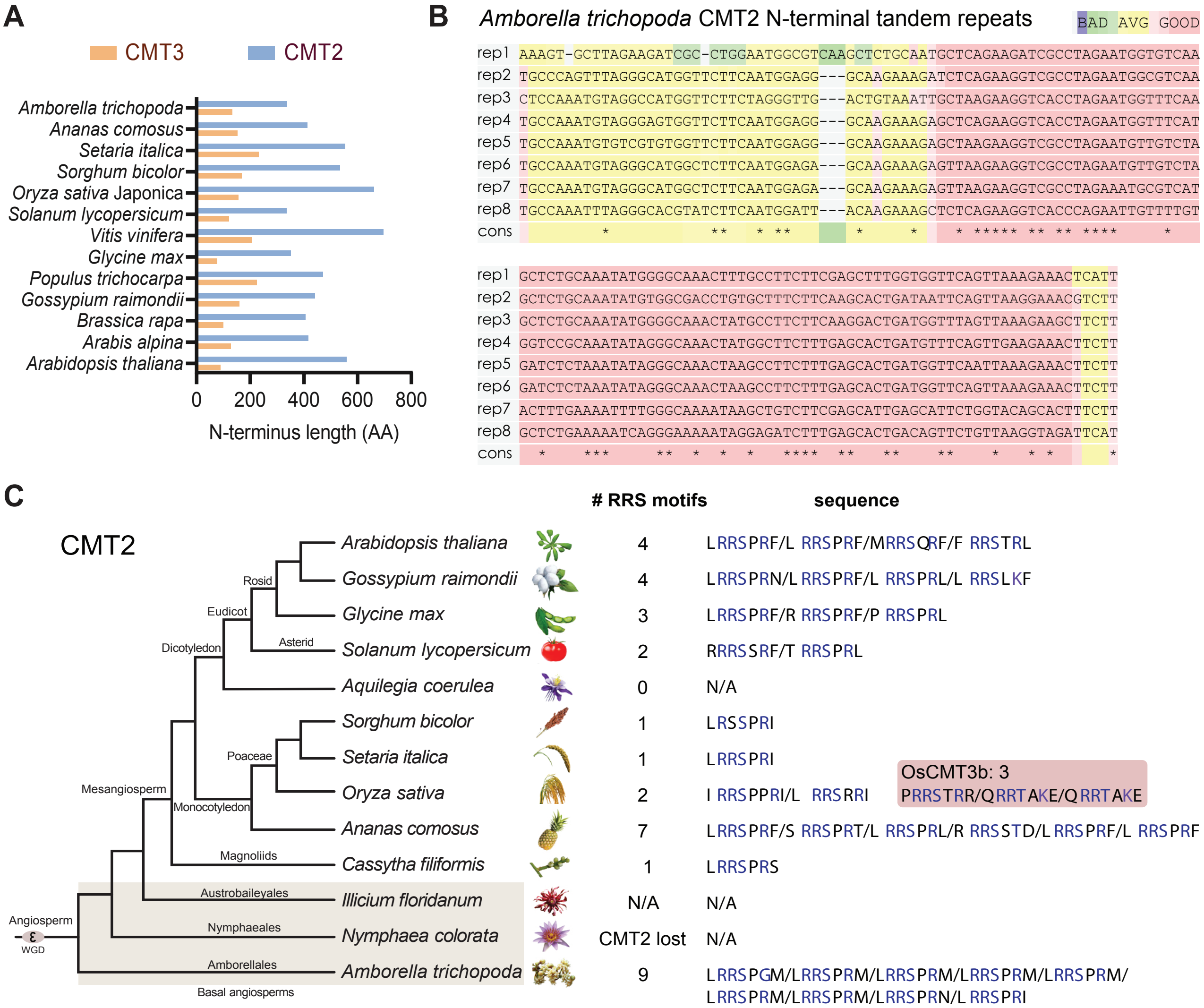

Figure S7. CMT2 RRS motifs are conserved in angiosperm species.
(A) N-terminus length of CMT3 and CMT2 from representative angiosperm species. (B) Alignment of the DNA sequences of eight tandem repeats in the N-terminus of *Amborella trichopoda* CMT2 by T-COFFEE. (C) The RRS motifs in CMT2 of representative angiosperm species.

A

```
pAmtriCMT3   TGGCGCGGACCAATTTTTCAGAATTCGGAGTACCCCCAGACACTCTCCTCCCTTGGATTATTAAATTCT
pAmtriCMT2   AGCCGCAGCCCAACTTGAATAAAAAGGG--------------CTGCAGCCCTATAGAAACTGAAACCAT
cons          * *** * ***** **     **   **              **     **  * **   * ***   *

pAmtriCMT3   GTTAATTTGTTCGAATGATTCCGCTTTCCCGCCCATCCCCG--------------CTCTTCGAAATCCC
pAmtriCMT2   AAAAGTTCAAGAGATAGGCCCCGCCTTAGCCAACACCGCAGTGCACTTTAACAAGCTGTCATTAAACTA
cons            * **     **  *   **** **  *   ** * * *              ** *    ** *

pAmtriCMT3   CCAAAGCACGATGTACACCCTTTCTCTCTCTAAACTATCTCTTTCTCTCTCTAAACATATATAAATACA
pAmtriCMT2   CAACCGTACGATCATATCTCTCCTTATTCGAACCGTCCATTCGACATCTCGTCTACTAACTCAAAAGAT
cons         * *  * *****     * **   * *    *   *   *    *      *  **  *   ***

pAmtriCMT3   ATCTTTTTTGTGCCCTATCTC----------TCTCCCTCTCTCATATTTTACAGTGGCCTCCCTCTCTC
pAmtriCMT2   AAGCGTTATATGCCAAAGTTCCACGCGTGAGTACCCCGCACGTATATATCCAACCTGGCATATTCATAT
cons         *    ** * ****  *  **          *  *** * *  **** *   *   * *    **

pAmtriCMT3   TCTCCTTCCCCTTCTCTCTCTCATTCAGGATTTCAGGTCACTGGAGCTCTTCTCCATTTGATTGAGCTC
pAmtriCMT2   TGCGCGCGCTCTCCTCAGCTGCATCCA------------------------CTGCGACTGAGAGAGTCC
cons         *   *   * ** ***     *** **                        ** *   ***  ***  *

pAmtriCMT3   TCTCTCTCTTGCCCTCTCCCTCTCTCTCTCTCTTGCCCTCTCCCTCTCTCTCTAGCTTCTGCTGTTCTT
pAmtriCMT2   CGGCGTCACTGCCCTTTTCTAGGGCAAAACTTTGCCCCCGTTTCTTCAAGATTAGGGCAAGTTTCTTCA
cons            *     ****** * *          ** *  ***  *  **        ***     * *  *

pAmtriCMT3   TGATCGACGAACCTTTCATTTTACCGAGCTTTTCCTATTTCCCCAATGTTTGAAGATTTTTCTATTTTA
pAmtriCMT2   ATGGAGGACCGTAAAGTGCTTAGAAGATCGCCTGGAATGGCGTCAAGCTCTGCAATGCTCAGAAGATCG
cons              *             **    ** *   *   **  *  ***  * ** *    *    *  *
                          repeat 1         motif 0                           motif 1

pAmtriCMT3   CTTGCATTCTAGCGTCCCTCTCTAGATATCTCGTTTTTGTGCCCTATCTCTTTCCTTTGATCTTTCATT
pAmtriCMT2   CCTAGAATGGTGTCAAGCTCTGCAAATATGGGGCAAACTTTGCCT-TCTTCGAGCTTTGGTGGTTCAGT
cons         * *  * *   *     ****  * ****   *      *  *** ***     ***** *  **** *

pAmtriCMT3   TACAGTGGCTTCTCTCTCGCTCATACTGGCTTCTATTAGGCACTGAAGCCCTTTTTCATTTGACTGGGC
pAmtriCMT2   TAAAGAAACTCATTTGCCCAGTTTAG-GGCATGGTTCTTCAATGGAGGGCAAGAAAGATCTCAGAAGGT
cons         ** **   **  * *  *     **  *** *   *     *  ** * *       ** * *   **
                            repeat 2                                          motif 2

pAmtriCMT3   TCTCTCTCTCTCTCTCAACAACTTCCACTGTTCTGTTGACGCTCCTTTCATTTGGCCGCGCTTTTTTGC
pAmtriCMT2   CGCCTAGAATGGCGTCAAGCTCTGCAAATATGTGGCGACCTGTGCTTTCTTCAAGCACTGATAATTCAG
cons            **         ****    ** * * * *   *    *  * ***** *   **   * *  **

pAmtriCMT3   CTTCCACAATTTCTGAAGATTTTTATCTATTGATTTTTCTATTTTCCTATAATTTACCTCTGATTGATT
pAmtriCMT2   TTAAGGAAACGTCT-------TCTCCAAATGTAGGCCATGGTTCTTCTAGGGTTGACTGTAAATTGCTA
cons          *     **  ***       * *    **  *        ** * ***   ** **     **** *
                                   repeat 3

pAmtriCMT3   TGTTGGGCCATTA-------------CCAATCAAAAAAGAAGAAAGATATTGTTTTTTTAGAGCCATTA
pAmtriCMT2   AGAAGGTCACCTAGAATGGTTTCAAGCTCTGCAAATATGGGGCAAACTATGCCTTCTTCAAGGACTGAT
cons          *  ** *   **             *    **** * *  * **  ***   ** ** *  * *
                  motif 3

pAmtriCMT3   TTTGCTTATTATTTACCTGCGATG
pAmtriCMT2   GGTTTAGTTAAAGAAGCTTCTTTG
cons           *     * *   * ** *  **
```

B

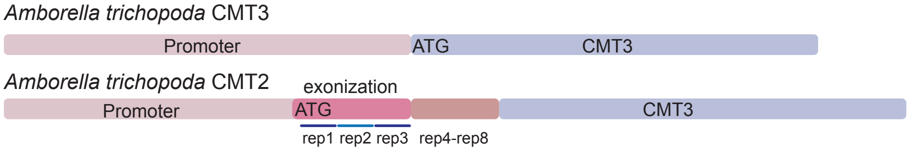

Figure S8. Alignment of *Amborella trichopoda* CMT2 and CMT3 promoter sequences.
(**A**) Alignment of the promoter sequences of AmtriCMT3 and AmtriCMT2. (**B**) Diagram showing the gene structure of AmtriCMT3 and AmtriCMT2.

A

Protein	Species	Predicted NLS	Score
CMT3	*Arabidopsis thaliana*	^{4}KRKRPATKDDTTKSIPKPKKRAPK^{27}	12.1
CMT2	*Arabidopsis thaliana*	^{176}GSRKRKSNSAL^{186}	6
		^{256}GSNKRMRRSQRF^{267}	5.5
CMT3	*Marchantia polymorpha*	^{76}RPTNRKRKSAPKVKAAAKRARKK^{98}	8.7
CMT3	*Chara braunii*	^{174}PPTKRQKPA^{182}	8.5
CMT2	*Amborella trichopoda*	^{381}YQRRVKKQKVG^{391}	7

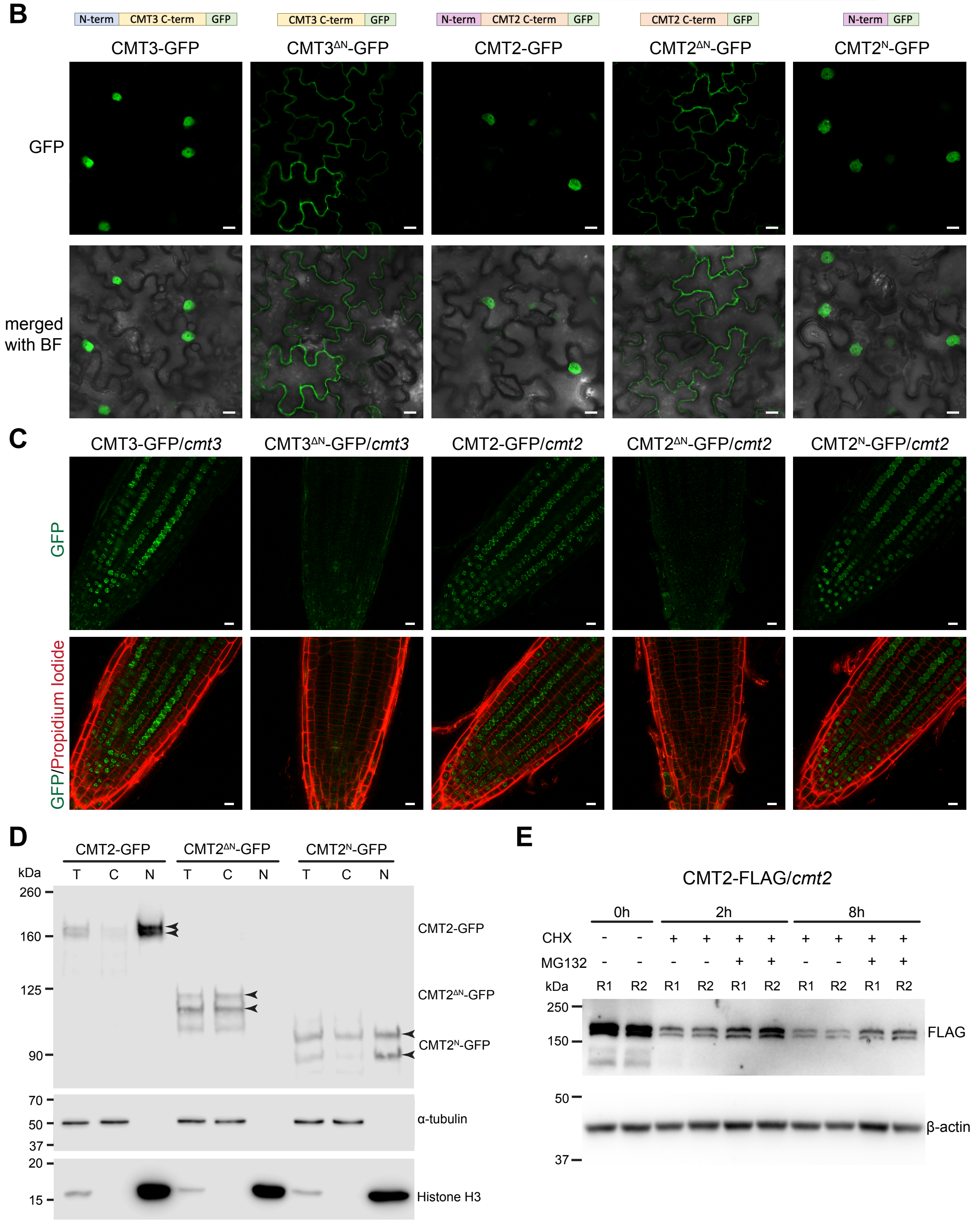

Figure S9. Nuclear localization signal dictates CMT2 and CMT3 localization.
(**A**) Predicted nuclear localization sequence in CMT2 and CMT3 of different plant species by NLS mapper (https://nls-mapper.iab.keio.ac.jp). (**B**) Images showing GFP signals in *N. benthamiana* leaves transiently expressed with indicated CMT3 or CMT2 protein (scale bar: 10 μm). (**C**) Images showing GFP signals in *Arabidopsis* root tip of indicated (scale bar: 10 μm). (**D**) Immunoblots showing CMT2 protein levels in different cellular fractions extracted from indicated transgenic seedlings. T: total, C: cytoplasmic, N: nuclear. (**E**) Immunoblots showing CMT2 protein level after cycloheximide (CHX, 500 μM) treatment with MG132 (50 μM) or DMSO for the indicated time. 7-day-old seedlings were used. Actin served as a control. R1 and R2 represent two biological replicates.

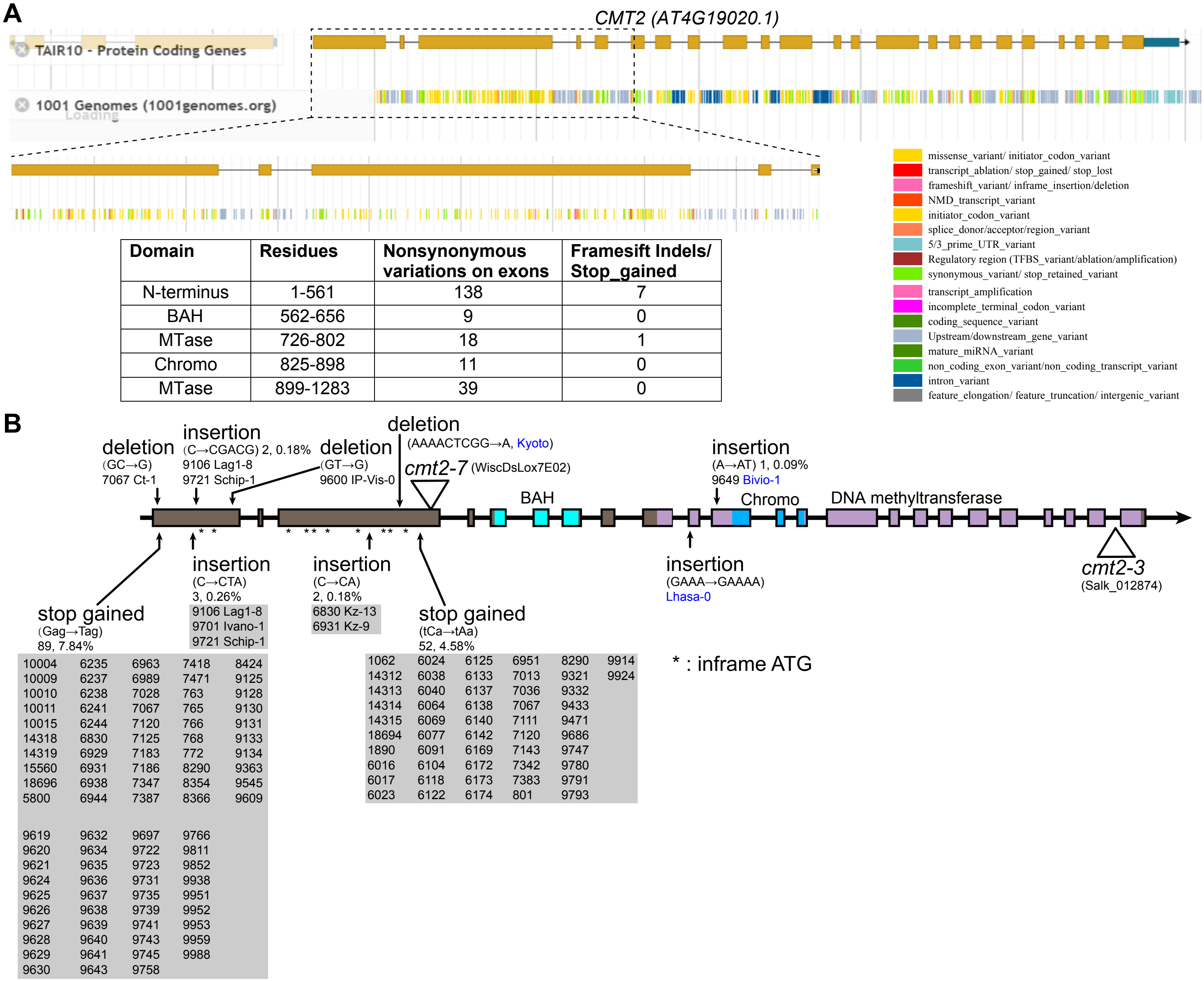

Domain	Residues	Nonsynonymous variations on exons	Framesift Indels/ Stop_gained
N-terminus	1-561	138	7
BAH	562-656	9	0
MTase	726-802	18	1
Chromo	825-898	11	0
MTase	899-1283	39	0

Figure S10. CMT2 natural variations from the 1001 genomes collection.
(**A**) Browser snapshot of CMT2 natural variations and number of exon nonsynonymous variations on CMT2 domains. The data were from TAIR Jbrowse (https://jbrowse.arabidopsis.org). (**B**) Diagram showing the details of frameshift-indels and stop-gained natural variations in CMT2. The ID of *Arabidopsis thaliana* natural accessions is from 1001 Genomes Project by Alonso-Blanco *et al.* (*31*) and can be found at https://wmd3.weigelworld.org/accessions.html. Kyoto is reported in Nozawa *et al.* (*27*). Lhasa-0 is from this study.
* indicates possible novel inframe translation start sites.

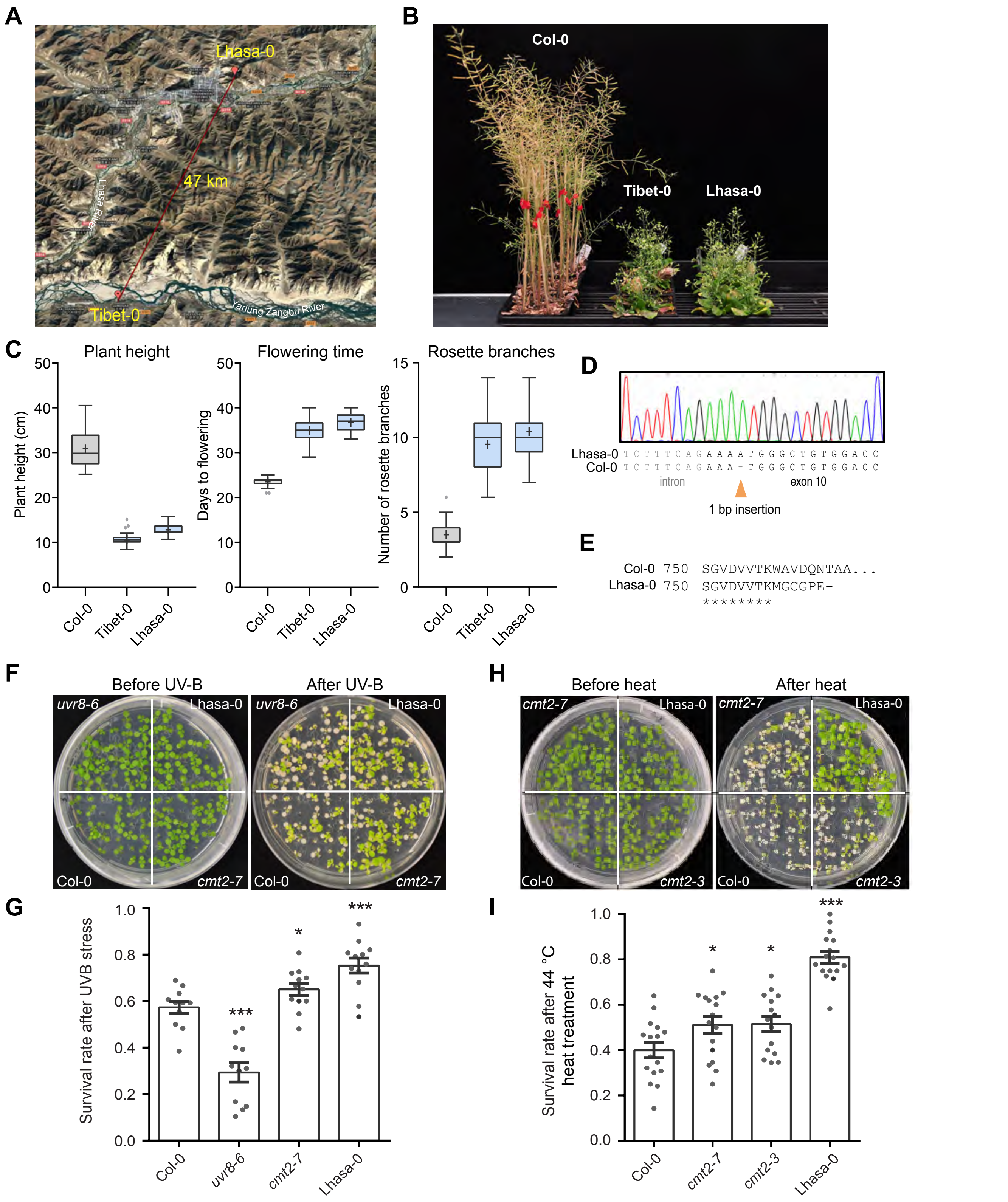

Figure S11. Characterization of *Arabidopsis thaliana* Lhasa-0 accession lacking functional CMT2.
(**A**) Image of regions where Tibet-0, discovered in Zeng *et al.* (*33*), and Lhasa-0 were isolated from the Tibetan Plateau near Lhasa. (**B**) Photos of Lhasa-0 at the flowering stage. (**C**) Phenotypes of plant height, flowering time, and rosette branches of Lhasa-0. Col-0 and Tibet-0 were used as controls. (**D**) Sanger sequencing confirming the 1 bp insertion in CMT2 of Lhasa-0. (**E**) Predicted amino acid sequence of the CMT2 mutation in Lhasa-0. (**F**) Images of seeding before and recovered from UV-B stress treatment. Treatment was performed for 3.5 hours on 10-day-old seedlings. (**G**) Survival rate in UV-B treatment from (F). Each grey dot represents a separate experimental replicate with n ≥ 25 plants. (**H**) Images of seedings before and recovered after 2 hours basal heat treatment (44°C). Treatment was performed on 10-day-old seedlings. (**I**) Survival rate of plants in basal heat stress from (H). Each grey dot represents a separate experimental replicate with n ≥ 25 plants.

A

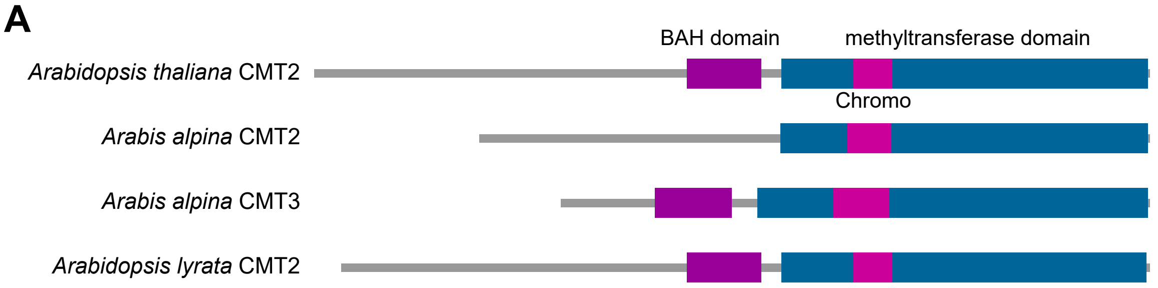

B

BAH domain

```
Arabis alpina CMT3    HIFELNDDAHVEAGVGVDPYICKIVEMFEGVDGELYFTAQWYYRAVDTVIELHETLINEK    205
Arabis alpina CMT2    QTFSLEDFAYI-K-----------------------------------------------    444
A. thaliana CMT2      HTFSLGDFAYI-KGEEEETHVGQIVEFFKTTDGESYFRVQWFYRATDTIMERQATNHDKR    636
A. lyrata CMT2        HTFSLGDFACI-KGEEEETHVGKILEFFKTTDEESYFRVQWFYRATDTVMKQQATNHDKK    594
                      : *.* * * :
                                                              ↑
                                                       H3K9me2 binding

Arabis alpina CMT3    RVFISEIRNANPLNTLVKKLNISRIPLMANAEETFAATRNCDYYCDTKYHLPYSTFEALL    265
Arabis alpina CMT2    ------------------------------------------------------------    444
A. thaliana CMT2      RLFYSTVMNDNPVDCLISKVTVLQVSPRVG---LKPNSIKSDYYFDMEYCVEYSTFQTLR    693
A. lyrata CMT2        RLFYSTVMNDNPVDCLISKVTVLQVSPMAG---LKPNSIKSDYYFDMEYCVEYSTFQTLR    651
```

C

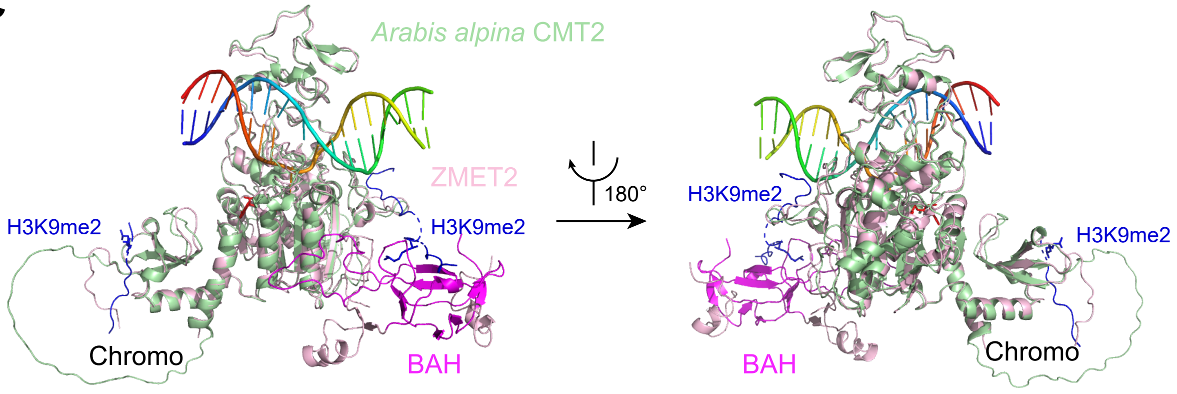

Figure S12. *Arabis alpina* CMT2 loses BAH domain.
(**A**) Domains of CMT2 from *Arabidopsis* and *Arabis alpina*. (**B**) Alignment of amino acids showing the loss of BAH domain in *Arabis alpina* CMT2. (C) Predicted structure of *Arabis alpina* CMT2 by AlphaFold in comparison with a ZMET2-DNA-H3K9me2 complex crystal structure in Fang *et al.* (*21*).

Table S1. Bisulfite sequencing statistics

Sample	Total reads	Aligned reads	% aligned	unique reads	% unique	*Fold Coverage	Bisulfite conversion
cmt3-11†	27076301	25452467	94.8	20279023	75.5	20.8	99.8
MpCMT3/*cmt3*, 4†	28014192	22740931	81.9	21648214	78.0	22.2	99.8
MpCMT3/*cmt3*, 5†	28209213	22409712	81.8	21087485	77.0	21.6	99.8
CbCMT3/*cmt3*, 9†	40763276	33755955	83.8	32348669	80.4	33.2	99.8
Col-0	59444216	56484853	95	44932612	75.6	18.9	99.6
cmt2cmt3	78761022	75925192	96.4	61503476	78.1	25.8	99.6
V1200R/*cc,*9	61468368	59093450	96.1	46876429	76.3	19.7	99.6
V1200R/*cc*, 15	72434966	69519000	96	55541358	76.7	23.3	99.6
CMT2/*cc*, 1	84464164	81582929	96.6	64840721	76.8	27.2	99.5
CMT2/*cc*, 19	62027070	59410963	95.8	47867980	77.2	20.1	99.6
ddcc	69397134	67056468	96.6	54650074	78.7	23	99.7
V1200R/*ddcc*, 3	76042607	73508084	96.7	58460949	76.9	24.6	99.6
V1200R/*ddcc*, 11	70703166	67427907	95.4	53712902	76	22.6	99.6
CMT2/*ddcc*, 27	77715525	74492342	95.9	58861516	75.7	24.7	99.6
CMT2/*ddcc,* 28	64558263	62159597	96.3	48970850	75.6	20.6	99.6
Tibet-0	29741378	17919404	60.3	8591803	28.9	10.8	96.1
Lhasa-0	31387665	16679257	53.1	7514174	23.9	9.5	98.2

*Fold coverage was calculated by multiplying the unique reads by the read length (pair of 61bp for †CMT3-related samples, 150 bp for Tibet-0 and Lhasa-0, and 50bp for all other samples) and dividing it by the size of the Arabidopsis genome (119Mb).

Table S2. RNA sequencing statistics

Sample	Raw reads	Trimmed reads	% aligned
Col-0, rep1	19236554	19235519	98.44%
Col-0, rep2	25349046	25348027	98.84%
cmt2cmt3, rep1	22662549	22661558	98.44%
cmt2cmt3, rep2	25007767	25006623	98.67%
CMT2/*cc*, 1, rep1	23210962	23209983	98.62%
CMT2/*cc*, 1, rep2	26373323	26372210	97.97%
CMT2/*cc*, 19, rep1	23368751	23367746	98.56%
CMT2/*cc*, 19, rep2	25041513	25040448	98.83%
V1200R/*cc*, 9, rep1	21943500	21942551	98.48%
V1200R/*cc*, 9, rep2	29942883	29941681	98.70%
V1200R/*cc*, 15, rep1	26491774	26490673	98.77%
V1200R/*cc*, 15, rep2	25112809	25111771	98.84%

Data S1. (separate file)
List of CMT3 and CMT2 protein sequences from various species.

Data S2. (separate file)
List of CMT2 natural variations.

Data S3. (separate file)
List of primers used in this study.

REFERENCES AND NOTES

1. A. L. Mattei, N. Bailly, A. Meissner, DNA methylation: A historical perspective. *Trends Genet.* **38**, 676–707 (2022).

2. R. J. Schmitz, Z. A. Lewis, M. G. Goll, DNA methylation: Shared and divergent features across eukaryotes. *Trends Genet.* **35**, 818–827 (2019).

3. M. V. C. Greenberg, D. Bourc'his, The diverse roles of DNA methylation in mammalian development and disease. *Nat. Rev. Mol. Cell Biol.* **20**, 590–607 (2019).

4. L. He, H. Huang, M. Bradai, C. Zhao, Y. You, J. Ma, L. Zhao, R. Lozano-Duran, J. K. Zhu, DNA methylation-free *Arabidopsis* reveals crucial roles of DNA methylation in regulating gene expression and development. *Nat. Commun.* **13**, 1335 (2022).

5. S. M. Leichter, J. Du, X. Zhong, Structure and mechanism of plant DNA methyltransferases. *Adv. Exp. Med. Biol.* **1389**, 137–157 (2022).

6. H. Zhang, Z. Lang, J. K. Zhu, Dynamics and function of DNA methylation in plants. *Nat. Rev. Mol. Cell Biol.* **19**, 489–506 (2018).

7. S. Henikoff, L. Comai, A DNA methyltransferase homolog with a chromodomain exists in multiple polymorphic forms in Arabidopsis. *Genetics* **149**, 307–318 (1998).

8. A. M. Lindroth, X. Cao, J. P. Jackson, D. Zilberman, C. M. McCallum, S. Henikoff, S. E. Jacobsen, Requirement of CHROMOMETHYLASE3 for maintenance of CpXpG methylation. *Science* **292**, 2077–2080 (2001).

9. A. J. Bewick, C. E. Niederhuth, L. Ji, N. A. Rohr, P. T. Griffin, J. Leebens-Mack, R. J. Schmitz, The evolution of CHROMOMETHYLASES and gene body DNA methylation in plants. *Genome Biol.* **18**, 65 (2017).

10. R. Ren, H. Wang, C. Guo, N. Zhang, L. Zeng, Y. Chen, H. Ma, J. Qi, Widespread whole genome duplications contribute to genome complexity and species diversity in angiosperms. *Mol. Plant* **11**, 414–428 (2018).

11. E. Kuzmin, J. S. Taylor, C. Boone, Retention of duplicated genes in evolution. *Trends Genet.* **38**, 59–72 (2022).

12. J. Du, X. Zhong, Y. V. Bernatavichute, H. Stroud, S. Feng, E. Caro, A. A. Vashisht, J. Terragni, H. G. Chin, A. Tu, J. Hetzel, J. A. Wohlschlegel, S. Pradhan, D. J. Patel, S. E. Jacobsen, Dual binding of chromomethylase domains to H3K9me2-containing nucleosomes directs DNA methylation in plants. *Cell* **151**, 167–180 (2012).

13. A. Zemach, M. Y. Kim, P. H. Hsieh, D. Coleman-Derr, L. Eshed-Williams, K. Thao, S. L. Harmer, D. Zilberman, The Arabidopsis nucleosome remodeler DDM1 allows DNA methyltransferases to access H1-containing heterochromatin. *Cell* **153**, 193–205 (2013).

14. H. Stroud, T. Do, J. Du, X. Zhong, S. Feng, L. Johnson, D. J. Patel, S. E. Jacobsen, Non-CG methylation patterns shape the epigenetic landscape in *Arabidopsis*. *Nat. struct. Mol. Biol.* **21**, 64–72 (2014).

15. J. M. Wendte, Y. Zhang, L. Ji, X. Shi, R. R. Hazarika, Y. Shahryary, F. Johannes, R. J. Schmitz, Epimutations are associated with CHROMOMETHYLASE 3-induced de novo DNA methylation. *Elife* **8**, e47891 (2019).

16. A. J. Bewick, L. Ji, C. E. Niederhuth, E. M. Willing, B. T. Hofmeister, X. Shi, L. Wang, Z. Lu, N. A. Rohr, B. Hartwig, C. Kiefer, R. B. Deal, J. Schmutz, J. Grimwood, H. Stroud, S. E. Jacobsen, K. Schneeberger, X. Zhang, R. J. Schmitz, On the origin and evolutionary consequences of gene body DNA methylation. *Proc. Natl. Acad. Sci. U.S.A.* **113**, 9111–9116 (2016).

17. K. Nozawa, J. Chen, J. Jiang, S. M. Leichter, M. Yamada, T. Suzuki, F. Liu, H. Ito, X. Zhong, DNA methyltransferase CHROMOMETHYLASE3 prevents ONSEN transposon silencing under heat stress. *PLOS Genet.* **17**, e1009710 (2021).

18. T. Kawakatsu, S.-S. C. Huang, F. Jupe, E. Sasaki, R. J. Schmitz, M. A. Urich, R. Castanon, J. R. Nery, C. Barragan, Y. He, H. Chen, M. Dubin, C.-R. Lee, C. Wang, F. Bemm, C. Becker, R. O'Neil, R. C. O'Malley, D. X. Quarless, 101 Genomes Consortium, N. J. Schork, D.

Weigel, M. Nordborg, J. R. Ecker, Epigenomic diversity in a global collection of Arabidopsis thaliana accessions. *Cell* **166**, 492–505 (2016).

19. L. He, C. Zhao, Q. Zhang, G. Zinta, D. Wang, R. Lozano-Duran, J. K. Zhu, Pathway conversion enables a double-lock mechanism to maintain DNA methylation and genome stability. *Proc. Natl. Acad. Sci. U.S.A.* **118**, e2107320118 (2021).

20. C. I. Stoddard, S. Feng, M. G. Campbell, W. Liu, H. Wang, X. Zhong, Y. Bernatavichute, Y. Cheng, S. E. Jacobsen, G. J. Narlikar, A nucleosome bridging mechanism for activation of a maintenance DNA methyltransferase. *Mol. Cell* **73**, 73–83.e6 (2019).

21. J. Fang, J. Jiang, S. M. Leichter, J. Liu, M. Biswal, N. Khudaverdyan, X. Zhong, J. Song, Mechanistic basis for maintenance of CHG DNA methylation in plants. *Nat. Commun.* **13**, 3877 (2022).

22. C. Noy-Malka, R. Yaari, R. Itzhaki, A. Mosquna, N. Auerbach Gershovitz, A. Katz, N. Ohad, A single CMT methyltransferase homolog is involved in CHG DNA methylation and development of Physcomitrella patens. *Plant Mol. Biol.* **84**, 719–735 (2014).

23. R. Yaari, A. Katz, K. Domb, K. D. Harris, A. Zemach, N. Ohad, RdDM-independent de novo and heterochromatin DNA methylation by plant CMT and DNMT3 orthologs. *Nat. Commun.* **10**, 1613 (2019).

24. C. Cheng, Y. Tarutani, A. Miyao, T. Ito, M. Yamazaki, H. Sakai, E. Fukai, H. Hirochika, Loss of function mutations in the rice chromomethylase OsCMT3a cause a burst of transposition. *Plant J.* **83**, 1069–1081 (2015).

25. Amborella Genome Project, The *Amborella* genome and the evolution of flowering plants. *Science* **342**, 1241089 (2013).

26. M. J. Dubin, P. Zhang, D. Meng, M. S. Remigereau, E. J. Osborne, F. Paolo Casale, P. Drewe, A. Kahles, G. Jean, B. Vilhjalmsson, J. Jagoda, S. Irez, V. Voronin, Q. Song, Q. Long, G. Ratsch, O. Stegle, R. M. Clark, M. Nordborg, DNA methylation in Arabidopsis has a genetic basis and shows evidence of local adaptation. *eLife* **4**, e05255 (2015).

27. K. Nozawa, S. Masuda, H. Saze, Y. Ikeda, T. Suzuki, H. Takagi, K. Tanaka, N. Ohama, X. Niu, A. Kato, H. Ito, Epigenetic regulation of ecotype-specific expression of the heat-activated transposon ONSEN. *Front. Plant Sci.* **13**, 899105 (2022).

28. E. Sasaki, T. Kawakatsu, J. R. Ecker, M. Nordborg, Common alleles of CMT2 and NRPE1 are major determinants of CHH methylation variation in Arabidopsis thaliana. *PLOS Genet.* **15**, e1008492 (2019).

29. X. Shen, J. De Jonge, S. K. G. Forsberg, M. E. Pettersson, Z. Sheng, L. Hennig, O. Carlborg, Natural CMT2 variation is associated with genome-wide methylation changes and temperature seasonality. *PLOS Genet* **10**, e1004842 (2014).

30. E. Sasaki, J. Gunis, I. Reichardt-Gomez, V. Nizhynska, M. Nordborg, Conditional GWAS of non-CG transposon methylation in *Arabidopsis thaliana* reveals major polymorphisms in five genes. *PLOS Genet.* **18**, e1010345 (2022).

31. The 1001 Genomes Consortium, 1,135 genomes reveal the global pattern of Polymorphism in Arabidopsis thaliana. *Cell* **166**, 481–491 (2016).

32. S. Cohen, L. Kramarski, S. Levi, N. Deshe, O. Ben David, E. Arbely, Nonsense mutation-dependent reinitiation of translation in mammalian cells. *Nucleic Acids Res.* **47**, 6330–6338 (2019).

33. L. Zeng, Z. Gu, M. Xu, N. Zhao, W. Zhu, T. Yonezawa, T. Liu, L. Qiong, T. Tersing, L. Xu, Y. Zhang, R. Xu, N. Sun, Y. Huang, J. Lei, L. Zhang, F. Xie, F. Zhang, H. Gu, Y. Geng, M. Hasegawa, Z. Yang, M. J. C. Crabbe, F. Chen, Y. Zhong, Discovery of a high-altitude ecotype and ancient lineage of Arabidopsis thaliana from Tibet. *Sci. Bull.* **62**, 1628–1630 (2017).

34. Y. Zhang, C. J. Harris, Q. Liu, W. Liu, I. Ausin, Y. Long, L. Xiao, L. Feng, X. Chen, Y. Xie, X. Chen, L. Zhan, S. Feng, J. J. Li, H. Wang, J. Zhai, S. E. Jacobsen, Large-scale comparative epigenomics reveals hierarchical regulation of non-CG methylation in *Arabidopsis*. *Proc. Natl. Acad. Sci. U.S.A* **115**, E1069–E1074 (2018).

35. G. Malik, M. Dangwal, S. Kapoor, M. Kapoor, Role of DNA methylation in growth and differentiation in Physcomitrella patens and characterization of cytosine DNA methyltransferases. *FEBS J.* **279**, 4081–4094 (2012).

36. Q. Gouil, D. C. Baulcombe, DNA methylation signatures of the plant chromomethyltransferases. *PLOS Genet.* **12**, e1006526 (2016).

37. T. Stuart, S. R. Eichten, J. Cahn, Y. V. Karpievitch, J. O. Borevitz, R. Lister, Population scale mapping of transposable element diversity reveals links to gene regulation and epigenomic variation. *eLife* **5**, e20777 (2016).

38. M. Zhang, J. Zhao, W. Y. Li, S. Q. Wen, H. L. Huang, J. Dong, B. Liu, G. Zhang, H. B. Wang, Y. T. Shen, H. L. Jin, Increased photosystem II translation efficiency as an important photoprotective mechanism in an *Arabidopsis thaliana* (Tibet-0) adapted to high light environments. *Environ Exp Bot* **183**, 104350 (2021).

39. E.-M. Willing, V. Rawat, T. Mandakova, F. Maumus, G. V. James, K. J. V. Nordstrom, C. Becker, N. Warthmann, C. Chica, B. Szarzynska, M. Zytnicki, M. C. Albani, C. Kiefer, S. Bergonzi, L. Castaings, J. L. Mateos, M. C. Berns, N. Bujdoso, T. Piofczyk, L. de Lorenzo, C. Barrero-Sicilia, I. Mateos, M. Piednoel, J. Hagmann, R. Chen-Min-Tao, R. Iglesias-Fernandez, S. C. Schuster, C. Alonso-Blanco, F. Roudier, P. Carbonero, J. Paz-Ares, S. J. Davis, A. Pecinka, H. Quesneville, V. Colot, M. A. Lysak, D. Weigel, G. Coupland, K. Schneeberger, Genome expansion of *Arabis alpina* linked with retrotransposition and reduced symmetric DNA methylation. *Nat. Plants* **1**, 14023 (2015).

40. G. Wos, R. R. Choudhury, F. Kolar, C. Parisod, Transcriptional activity of transposable elements along an elevational gradient in Arabidopsis arenosa. *Mob. DNA* **12**, 7 (2021).

41. J. Jiang, J. Liu, D. Sanders, S. Qian, W. Ren, J. Song, F. Liu, X. Zhong, UVR8 interacts with de novo DNA methyltransferase and suppresses DNA methylation in *Arabidopsis*. *Nat. Plants* **7**, 184–197 (2021).

42. L. Quadrana, A. Bortolini Silveira, G. F. Mayhew, C. LeBlanc, R. A. Martienssen, J. A. Jeddeloh, V. Colot, The Arabidopsis thaliana mobilome and its impact at the species level. *eLife* **5**, (2016).

43. T. Westerhold, N. Marwan, A. J. Drury, D. Liebrand, C. Agnini, E. Anagnostou, J. S. K. Barnet, S. M. Bohaty, D. De Vleeschouwer, F. Florindo, T. Frederichs, D. A. Hodell, A. E. Holbourn, D. Kroon, V. Lauretano, K. Littler, L. J. Lourens, M. Lyle, H. Palike, U. Rohl, J. Tian, R. H. Wilkens, P. A. Wilson, J. C. Zachos, An astronomically dated record of Earth's climate and its predictability over the last 66 million years. *Science* **369**, 1383–1387 (2020).

44. T. L. Shimada, T. Shimada, I. Hara-Nishimura, A rapid and non-destructive screenable marker, FAST, for identifying transformed seeds of Arabidopsis thaliana. *Plant J.* **61**, 519–528 (2010).

45. X. Chen, L. Lu, K. S. Mayer, M. Scalf, S. Qian, A. Lomax, L. M. Smith, X. Zhong, POWERDRESS interacts with HISTONE DEACETYLASE 9 to promote aging in *Arabidopsis*. *Elife* **5**, e17214 (2016).

46. J. Chen, J. Liu, J. Jiang, S. Qian, J. Song, R. Kabara, I. Delo, G. Serino, F. Liu, Z. Hua, X. Zhong, F-box protein CFK1 interacts with and degrades de novo DNA methyltransferase in Arabidopsis. *New Phytol.* **229**, 3303–3317 (2021).

47. S. Chen, Y. Zhou, Y. Chen, J. Gu, fastp: An ultra-fast all-in-one FASTQ preprocessor. *Bioinformatics* **34**, i884–i890 (2018).

48. Y. Xi, W. Li, BSMAP: Whole genome bisulfite sequence MAPping program. *BMC Bioinformatics* **10**, 232 (2009).

49. A. Akalin, M. Kormaksson, S. Li, F. E. Garrett-Bakelman, M. E. Figueroa, A. Melnick, C. E. Mason, methylKit: A comprehensive R package for the analysis of genome-wide DNA methylation profiles. *Genome Biol.* **13**, R87 (2012).

50. A. R. Quinlan, I. M. Hall, BEDTools: A flexible suite of utilities for comparing genomic features. *Bioinformatics* **26**, 841–842 (2010).

51. F. Ramirez, D. P. Ryan, B. Gruning, V. Bhardwaj, F. Kilpert, A. S. Richter, S. Heyne, F. Dundar, T. Manke, deepTools2: A next generation web server for deep-sequencing data analysis. *Nucleic Acids Res.* **44**, W160–W165 (2016).

52. D. Kim, J. M. Paggi, C. Park, C. Bennett, S. L. Salzberg, Graph-based genome alignment and genotyping with HISAT2 and HISAT-genotype. *Nat Biotechnol* **37**, 907–915 (2019).

53. M. Pertea, G. M. Pertea, C. M. Antonescu, T. C. Chang, J. T. Mendell, S. L. Salzberg, StringTie enables improved reconstruction of a transcriptome from RNA-seq reads. *Nat. Biotechnol.* **33**, 290–295 (2015).

54. M. I. Love, W. Huber, S. Anders, Moderated estimation of fold change and dispersion for RNA-seq data with DESeq2. *Genome Biol.* **15**, 550 (2014).

55. H. Mi, A. Muruganujan, X. Huang, D. Ebert, C. Mills, X. Guo, P. D. Thomas, Protocol update for large-scale genome and gene function analysis with the PANTHER classification system (v.14.0). *Nat. Protoc.* **14**, 703–721 (2019).

56. B. Langmead, S. L. Salzberg, Fast gapped-read alignment with Bowtie 2. *Nat. Methods* **9**, 357–359 (2012).

57. H. Li, B. Handsaker, A. Wysoker, T. Fennell, J. Ruan, N. Homer, G. Marth, G. Abecasis, R. Durbin, 1000 Genome Project Data Processing Subgroup, The Sequence Alignment/Map format and SAMtools. *Bioinformatics* **25**, 2078–2079 (2009).

58. Y. Zhang, T. Liu, C. A. Meyer, J. Eeckhoute, D. S. Johnson, B. E. Bernstein, C. Nusbaum, R. M. Myers, M. Brown, W. Li, X. S. Liu, Model-based analysis of ChIP-Seq (MACS). *Genome Biol.* **9**, R137 (2008).

59. B. Xue, R. L. Dunbrack, R. W. Williams, A. K. Dunker, V. N. Uversky, PONDR-FIT: a meta-predictor of intrinsically disordered amino acids. *Biochim Biophys Acta* **1804**, 996-1010 (2010).

60. A. M. Bolger, M. Lohse, B. Usadel, Trimmomatic: A flexible trimmer for Illumina sequence data. *Bioinformatics* **30**, 2114–2120 (2014).

61. K. Wang, M. Li, H. Hakonarson, ANNOVAR: Functional annotation of genetic variants from high-throughput sequencing data. *Nucleic Acids Res.* **38**, e164 (2010).

62. H. Wickham, “Use R!” in *Ggplot2: Elegant Graphics for Data Analysis* (Springer, 1st ed., 2009).

63. H. Wickham, Reshaping data with the reshape package. *J. Stat. Softw.* **21**, 1–20 (2007).

64. A. J. Bewick, R. J. Schmitz, Epigenetics in the wild. *eLife* **4**, e07808 (2015).

65. S. Kumar, G. Stecher, K. Tamura, MEGA7: Molecular evolutionary genetics analysis version 7.0 for bigger datasets. *Mol. Biol. Evol.* **33**, 1870–1874 (2016).

66. S. L. K. Pond, S. D. W. Frost, S. V. Muse, HyPhy: Hypothesis testing using phylogenies. *Bioinformatics* **21**, 676–679 (2005).

67. S. Kryazhimskiy, J. B. Plotkin, The population genetics of dN/dS. *PLOS Genet* **4**, e1000304 (2008).

68. Y. Liu, T. Tian, K. Zhang, Q. You, H. Yan, N. Zhao, X. Yi, W. Xu, Z. Su, PCSD: A plant chromatin state database. *Nucleic Acids Res.* **46**, D1157–D1167 (2018).
